# Identification of Transcriptomic Differences between Lower Extremities Arterial Disease, Abdominal Aortic Aneurysm and Chronic Venous Disease in Peripheral Blood Mononuclear Cells Specimens

**DOI:** 10.3390/ijms22063200

**Published:** 2021-03-21

**Authors:** Daniel P. Zalewski, Karol P. Ruszel, Andrzej Stępniewski, Dariusz Gałkowski, Jacek Bogucki, Przemysław Kołodziej, Jolanta Szymańska, Bartosz J. Płachno, Tomasz Zubilewicz, Marcin Feldo, Janusz Kocki, Anna Bogucka-Kocka

**Affiliations:** 1 Chair and Department of Biology and Genetics, Medical University of Lublin, 4a Chodźki St., 20-093 Lublin, Poland; anna.kocka@umlub.pl; 2 Chair of Medical Genetics, Department of Clinical Genetics, Medical University of Lublin, 11 Radziwiłłowska St., 20-080 Lublin, Poland; karol.ruszel@umlub.pl (K.P.R.); janusz.kocki@umlub.pl (J.K.); 3 Ecotech Complex Analytical and Programme Centre for Advanced Environmentally Friendly Technologies, University of Marie Curie-Skłodowska, 39 Głęboka St., 20-612 Lublin, Poland; andrzej.stepniewski@umcs.pl; 4 Department of Pathology and Laboratory Medicine, Rutgers-Robert Wood Johnson Medical School, One Robert Wood Johnson Place, New Brunswick, NJ 08903-0019, USA; galkowd@fastmail.fm; 5 Chair and Department of Organic Chemistry, Medical University of Lublin, 4a Chodźki St., 20-093 Lublin, Poland; jacek.bogucki@umlub.pl; 6 Laboratory of Diagnostic Parasitology, Chair and Department of Biology and Genetics, Medical University of Lublin, 4a Chodźki St., 20-093 Lublin, Poland; przemyslaw.kolodziej@umlub.pl; 7 Department of Integrated Paediatric Dentistry, Chair of Integrated Dentistry, Medical University of Lublin, 6 Chodźki St., 20-093 Lublin, Poland; szymanska.polska@gmail.com; 8 Department of Plant Cytology and Embryology, Institute of Botany, Faculty of Biology, Jagiellonian University in Kraków, 9 Gronostajowa St., 30-387 Cracow, Poland; bartosz.plachno@uj.edu.pl; 9 Chair and Department of Vascular Surgery and Angiology, Medical University of Lublin, 11 Staszica St., 20-081 Lublin, Poland; tomasz.zubilewicz@umlub.pl (T.Z.); martinf@interia.pl (M.F.)

**Keywords:** lower extremities arterial disease, chronic venous disease, abdominal aortic aneurysm, gene expression, next generation sequencing, biomarker, transcriptome profiling

## Abstract

Several human tissues are investigated in studies of molecular biomarkers associated with diseases development. Special attention is focused on the blood and its components due to combining abundant information about systemic responses to pathological processes as well as high accessibility. In the current study, transcriptome profiles of peripheral blood mononuclear cells (PBMCs) were used to compare differentially expressed genes between patients with lower extremities arterial disease (LEAD), abdominal aortic aneurysm (AAA) and chronic venous disease (CVD). Gene expression patterns were generated using the Ion S5XL next-generation sequencing platform and were analyzed using DESeq2 and UVE-PLS methods implemented in R programming software. In direct pairwise analysis, 21, 58 and 10 differentially expressed genes were selected from the comparison of LEAD vs. AAA, LEAD vs. CVD and AAA vs. CVD patient groups, respectively. Relationships between expression of dysregulated genes and age, body mass index, creatinine levels, hypertension and medication were identified using Spearman rank correlation test and two-sided Mann–Whitney U test. The functional analysis, performed using DAVID website tool, provides potential implications of selected genes in pathological processes underlying diseases studied. Presented research provides new insight into differences of pathogenesis in LEAD, AAA and CVD, and selected genes could be considered as potential candidates for biomarkers useful in diagnosis and differentiation of studied diseases.

## 1. Introduction

Noncommunicable diseases such as cancer, diabetes mellitus, cardiovascular and chronic respiratory diseases are today the major contributors to the global burden of death instances [1,2]. Out of them conditions linked to cardiovascular system are one of the most common cause of death worldwide and their contribution will be even higher in future decades [1,3]. This work encompasses comparative gene expression analysis of three conditions from this vast group of diseases: lower extremities arterial disease (LEAD), abdominal aortic aneurysm (AAA) and chronic venous disease (CVD), which are complex and multifactorial vascular diseases burdened with high prevalence and severe life-threating consequences, making them major global health problems.

LEAD is the most common manifestation of peripheral arterial disease (PAD), characterized by chronic degenerative changes due to vascular flow deficit caused by formation of atheromatous plaques in arteries of lower limbs [4,5]. Overall global prevalence of LEAD is estimated to 5.56% of individuals aged 25 years and older and exceeds 10% in people older than 70 years [6]. The risk factors for LEAD include age, dyslipidemia, diabetes, smoking, hypertension and cardiovascular disease history [4,6]. The typical symptom of LEAD is intermittent claudication, resulting from recurrent ischemia-reperfusion cycles during physical activity, however more than 50% of LEAD cases is asymptomatic [5,7]. One of the most severe complication of LEAD is chronic limb-threatening ischemia, which affects approximately 11% of patients with LEAD [4,8].

AAA is a focal dilatation of the abdominal aorta measuring 50% greater than the proximal normal segment, or >3 cm in maximum diameter [9]. The prevalence of AAA ranges between 4% and 8% in general population of men aged 65–80 years [10]. Identified risk factors for aneurysm development include older age, male gender, cigarette smoking, obesity, dysregulation of lipid levels, hypertension [9,11,12] and genetic predisposition [13,14]. Patients with AAA may report nonspecific symptoms such as abdominal and back pain; however, in many cases, disease progress is asymptomatic [15]. Globally, AAA rupture is responsible for 0.3–0.4% of all death causes and approximately 1% of deaths among men above 65 years [16]. AAA rupture is associated with high distensibility of aortic wall, higher peak wall stress and aortic calcification [17].

CVD is defined as a syndrome of chronic morphological and functional abnormalities of the venous system caused by venous wall remodeling related to vascular inflammation, leading to venous hypertension, venous valve incompetency and reflux in veins of lower limbs [18,19,20,21]. The disease encompasses a wide spectrum of clinical presentations such as telangiectasia, varicose veins, leg edema, skin changes and ulcers [22]. The prevalence of symptomatic CVD among general practitioner attendees was estimated as high as 60%. The common risk factors include age, obesity, low physical activity, periods of prolonged standing or sitting and positive family history [23].

Guidelines for LEAD, AAA and CVD management, accomplished by specialists in the field and regularly updated [4,9,20,24,25], indicate urgent need for more effective diagnostic, treatment and differentiating strategies. Despite different clinical onset of LEAD, AAA and CVD, these diseases share main pathological mechanisms, such as inflammation, endothelial dysfunction and vascular smooth muscle cells proliferation and apoptosis, potentially impeding identification of specific biomarkers able to distinguish individuals affected with LEAD, AAA and CVD. Elucidation of molecular aspects of these diseases, including alterations in gene expression patterns associated with vascular pathology, could provide more focused insight into pathological conditions governing variety of vascular diseases.

Many studies have identified dysregulations of gene expression associated with vascular cell functions, including cell differentiation, proliferation, migration, and apoptosis exhibiting modulatory function of angiogenesis, endothelial cells dysfunction and response for ischemic events and oxidative stress [26,27,28,29,30,31]. Alterations in expression of numerous genes are considered as potential signatures of vascular diseases including atherosclerosis [32,33,34], LEAD [35,36,37], AAA [38,39,40,41,42] and CVD [43,44,45,46].

Peripheral blood mononuclear cells (PBMCs) represent a white blood cell subpopulation that includes lymphocytes and monocytes and constitute blood-derived clinical material broadly studied for elucidation of diseases processes due to possessing abundant information about systemic alterations in pathology. High accessibility of PBMCs make them a valuable source of potential biomarkers useful for detection and monitoring of disease progress. Changes in transcriptome expression of PBMCs may reflect ongoing local pathological processes in vascular tissues in either LEAD, AAA and CVD. Moreover, PBMCs subpopulations are key regulators of vascular inflammation, which is an important element of either LEAD, AAA or CVD onsets.

Therefore, transcriptomic patterns of PBMCs subpopulations originating from patients with LEAD, AAA and CVD versus healthy subjects were previously compared and potential transcriptomic biomarkers of these diseases were proposed [37,42,46]. In the current study, pairwise comparison of gene expression profiles between LEAD, AAA and CVD were performed in order to identify transcriptomic similarities and differences of these conditions. Obtained results could provide deeper insight into either general mechanisms of vascular pathology or unique processes contributing to LEAD, AAA and CVD. Identification of specific biomarkers potentially enables to select and monitor patients with high cardiovascular risk and to classify affected individuals to LEAD, AAA and CVD group, providing new diagnostic and treatment perspectives.

The study design, methodology and language were inspired by our previous studies regarding dysregulation of microRNA (miRNA) regulatory network in LEAD [37], AAA [42] and CVD [46] compared to healthy controls.

## 2. Results

### 2.1. Study Group Characteristics

The study group included 8 patients with LEAD, 7 patients with AAA and 7 patients with CVD. Clinical characteristics of participants are presented in Table 1 and detailed description of clinical features specific for each disease was provided in Table A1 in Appendix A. Statistical analysis of studied groups showed statistically significant (*p* < 0.05) differences in some characteristics, including age, BMI (body mass index), smoking habits, hypertension status, creatinine serum level and medication with statins, acetylsalicylic acid and beta-adrenergic blockers. These differences are a result of different risk factors and medication related to studied diseases. The influence of demographical and clinical differences on obtained results was examined and discussed further in the text.

### 2.2. The Comparison of Differentially Expressed Genes in PBMCs of LEAD, AAA and CVD Subjects in Relation to Healthy Controls

In our previous studies, whole transcriptome and miRNA expression profiles of PBMCs in patients with LEAD [37], AAA [42] and CVD [46] in relation to healthy controls were investigated. MiRNAs and genes with the most promising biomarker potential as well as alterations of miRNA regulatory network associated with analyzed diseases were identified. Further functional analyzes draw interesting relations between proposed biomarkers and the etiopathology of studied diseases.

**Figure 1:**
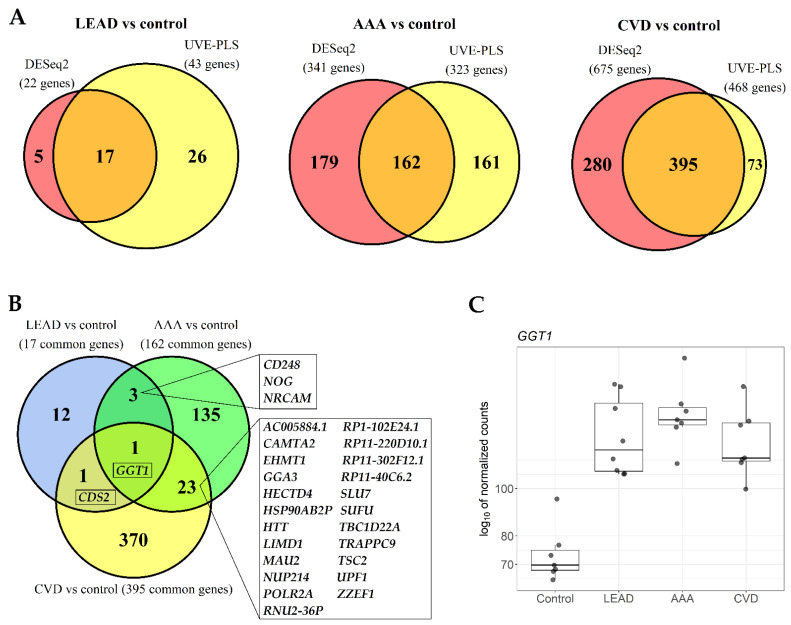
The comparison of differentially expressed genes in lower extremities arterial disease (LEAD), abdominal aortic aneurysm (AAA) and chronic venous disease (CVD) in relation to healthy controls. (**A**) The comparisons of differentially expressed genes in LEAD vs. control, AAA vs. control and CVD vs. control comparisons. Genes were obtained using DESeq2 and UVE-PLS methods and unified selection criteria (*p* value adjusted by Benjamini–Hochberg false discovery rate < 0.001 and reliability score ≥ 8, respectively). The numbers in the middle fields in each Venn diagram represent the amount of genes common for both methods. (**B**) The comparison of the genes common for DESeq2 and UVE-PLS methods from the Venn diagrams on panel A. The rectangles present gene symbols located in the corresponding fields of the Venn diagram. (**C**) Boxplot for the log10 of normalized counts of *GGT1*, which was found as a common gene on the Venn diagram from panel B. Whiskers define range between minimum and maximum value, boxes range between 25% and 75% quartile, horizontal lines inside boxes mark median value. UVE-PLS-Uninformative Variable Elimination by Partial Least Squares.

In this paper, we continue our investigations on molecular aspects of LEAD, AAA and CVD etiopathogenesis by searching for transcriptomic differences and similarities between these diseases. In the first approach, we compared previously obtained results of differential expression analysis that were performed for the total number of 55,765 genes using DESeq2 and UVE-PLS (Uninformative Variable Elimination by Partial Least Squares) methods between disease groups in the background of healthy controls (LEAD vs. control, AAA vs. control and CVD vs. control) [37,42,46]. To reduce and to assure high comparability of the data, strict and unified cutoff criteria for gene selection were applied: for DESeq2 results—genes with *p* value (adjusted by Benjamini–Hochberg false discovery rate) below 0.001 were selected and for UVE-PLS results—genes with the minimum reliability score equal to 8 were selected. Application of *p* < 0.001 threshold to DESeq2 data resulted in a selection of 22 differentially expressed genes in LEAD vs. control, 341 differentially expressed genes in AAA vs. control and 675 differentially expressed genes in CVD vs. control. As a result of establishing reliability score ≥8 as a selection criterium of differentially expressed genes resulted from UVE-PLS, 43 genes in LEAD vs. control, 323 genes in AAA vs. control and 468 genes in CVD vs. control were obtained. The sets of selected genes were compared within each studied disease on Venn diagrams and 17, 162 and 395 genes were obtained as common for both methods for LEAD vs. control, AAA vs. control and CVD vs. control, respectively (Figure 1A). Differential expression characteristics of common genes, including adjusted *p* values, fold changes and Partial Least Squares (PLS) coefficients are provided in Supplementary File 2.

To identify transcriptomic similarities and differences between LEAD, AAA and CVD, the sets of selected 17, 162 and 395 genes were compared on Venn diagram (Figure 1B). Differential expression of one gene (*GGT1*, gamma-glutamyltransferase 1) was common for all analyzed gene sets, one gene (*CDS2*, CDP-diacylglycerol synthase 2) was common for LEAD vs. control and CVD vs. control, three genes were common for LEAD vs. control and AAA vs. control, and 23 genes were common for AAA vs. control and CVD vs. control (Figure 1B). To conclude, the approach of making comparisons of differentially expressed gene sets in LEAD, AAA and CVD in relation to control group indicated that upregulation of *GGT1* could be considered as a common feature of PBMCs from patients with these diseases (Figure 1C). Moreover, the differential expression of 12, 135 and 370 genes are specific for the comparisons LEAD vs. control, AAA vs. control and CVD vs. control, respectively (Figure 1B). These genes were additionally highlighted in the Supplementary File 2.

**Figure 2:**
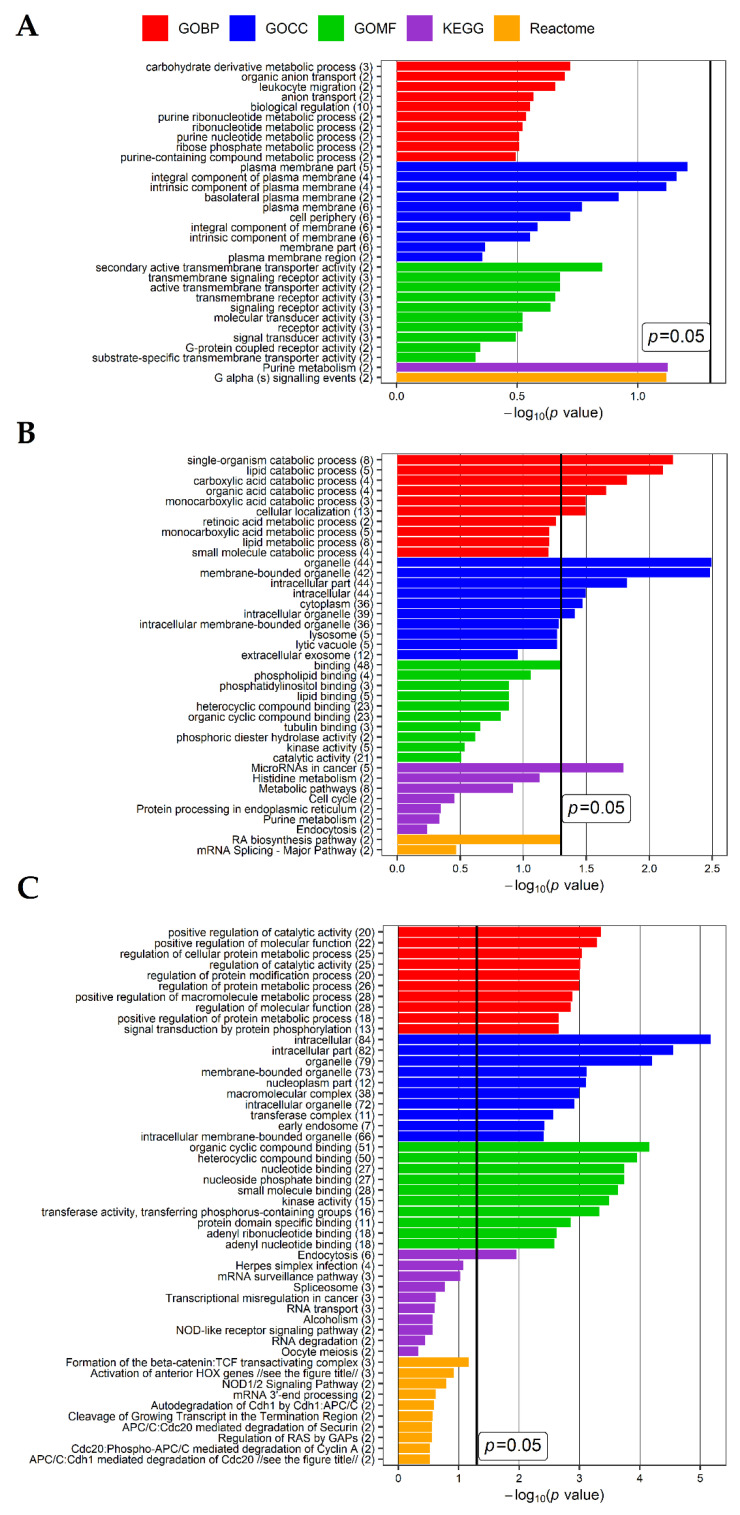
Results of functional analysis performed using DAVID website tool for the sets of (**A**) 12 potentially specific genes for the comparison of lower extremities arterial disease (LEAD) vs. control, (**B**) 135 potentially specific genes for the comparison of abdominal aortic aneurysm (AAA) vs. control and (**C**) 370 potentially specific genes for the comparison of chronic venous disease (CVD) vs. control (refer to panel B on the Figure 1). Up to top ten the most enriched terms of Gene Ontology Biological Processing (GOBP), Gene Ontology Cellular Compartment (GOCC), Gene Ontology Molecular Function (GOMF), KEGG (Kyoto Encyclopedia of Genes and Genomes) and Reactome categories were presented. *p* value—EASE score for enrichment, the thick black vertical line represents *p* = 0.05 threshold. The numbers in brackets following the names of terms indicate the numbers of associated genes. Due to a large length of some names of Reactome terms on panel C, to make the figure more readable they were shown in shorten form: the full name of “APC/C:Cdh1 mediated degradation of Cdc20” term is “APC/C:Cdh1 mediated degradation of Cdc20 and other APC/C:Cdh1 targeted proteins in late mitosis/early G1”, the full name of “Activation of anterior HOX genes” term is “Activation of anterior HOX genes in hindbrain development during early embryogenesis”.

To further examine differences between diseases, the potential biological role of 12, 135 and 370 genes specific for LEAD vs. control, AAA vs. control and CVD vs. control comparisons, respectively, was determined in functional enrichment analysis performed using Database for Annotation, Visualization and Integrated Discovery (DAVID) database. Up to top ten enriched terms (with the lowest *p* value of enrichment) of Gene Ontology Biological Processing (GOBP), Gene Ontology Cellular Compartment (GOCC), Gene Ontology Molecular Function (GOMF), KEGG (Kyoto Encyclopedia of Genes and Genomes) and Reactome categories for each gene sets were selected and presented on Figure 2.

### 2.3. The Comparison of Differentially Expressed Genes in PBMCs of LEAD, AAA and CVD Subjects after Direct, Pairwise Comparisons

**Figure 3:**
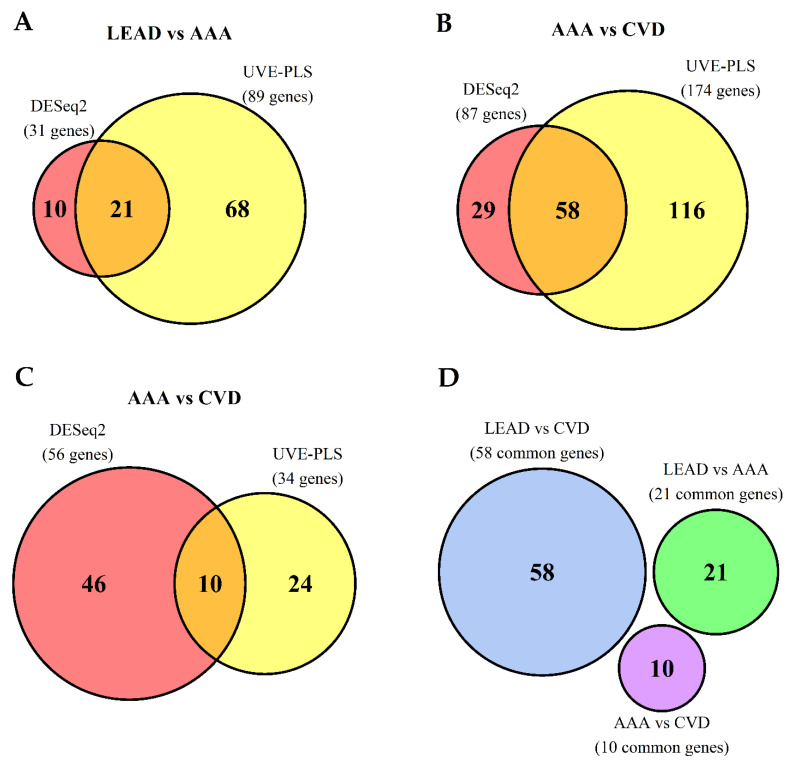
The comparison of differentially expressed genes resulted from comparative analysis performed using DESeq2 and UVE-PLS methods between (**A**) LEAD and AAA groups, (**B**) LEAD and CVD groups as well as (**C**) AAA and CVD groups. Genes were selected using unified selection criteria: *p* value adjusted by Benjamini–Hochberg false discovery rate < 0.001 (for DESeq2 results) and reliability score ≥ 8 (for UVE-PLS results). The number in the middle fields in each Venn diagram represents the number of genes common for both methods. Genes shared by both methods from each comparison (genes from the middle fields in Venn diagrams on panel **A**–**C**) were selected and compared on the subsequent Venn diagram (**D**), which shows a lack of sharing genes. AAA—abdominal aortic aneurysm, CVD—chronic venous disease, LEAD—lower extremities arterial disease, UVE-PLS—Uninformative Variable Elimination by Partial Least Squares.

To further investigate transcriptomic similarities and differences in PBMCs of LEAD, AAA and CVD subjects, a direct, pairwise differential gene expression analysis was performed within these groups. In this approach, LEAD vs. AAA, LEAD vs. CVD and AAA vs. CVD comparisons were performed using DESeq2 and UVE-PLS methods. To assess the quality of the data, control plots including MA plot and histogram of *p* values were generated and evaluated for each comparison (Supplementary Materials Figures S1–S3). The boxplot of Cook’s distances of genes across all samples presents lack of any outliers in analyzed data (Figure S4). Similar to the previous approach, the unified cutoff thresholds (corrected *p* < 0.001 and reliability score ≥8) were used for gene selection from DESeq2 and UVE-PLS results, respectively (Table A2). Sets of genes selected from used methods were compared on Venn diagrams, revealing 21 genes (9 upregulated and 12 downregulated) common for DESeq2 and UVE-PLS methods from the comparison of LEAD vs. AAA (Figure 3A), 58 genes (43 upregulated and 15 downregulated) common for both methods from the comparison of LEAD vs. CVD (Figure 3B) and 10 genes (all downregulated) common for both methods from the comparison of AAA vs. CVD (Figure 3C, Table 2).

**Figure 4:**
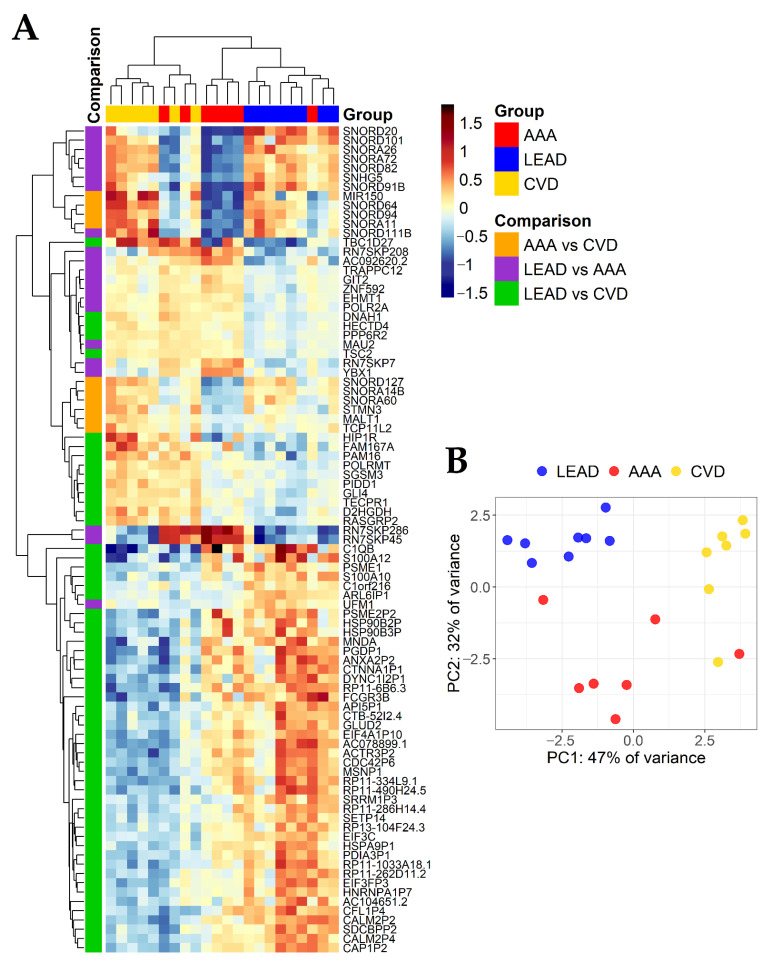
Differential expression of 21, 58 and 10 genes identified as unique for LEAD vs. AAA, LEAD vs. CVD and AAA vs. CVD comparisons, respectively. (**A**) Heatmap with clustering of Euclidean distances using complete method. (**B**) Principal Component Analysis (PCA) plot. LEAD—lower extremities arterial disease, AAA—abdominal aortic aneurysm, CVD—chronic venous disease.

To identify common and unique genes for LEAD vs. AAA, LEAD vs. CVD and AAA vs. CVD diseases pairs, the obtained sets of 21, 58 and 10 genes were compared on the subsequent Venn diagram (Figure 3D), which shows a lack of genes shared by all three sets of genes. It indicates that these gene sets could be considered as potentially unique for performed comparisons and presumably useful to differentiate studied diseases. Differential expression characteristics of 89 (21 + 58 + 10) unique genes, including adjusted *p* values, fold changes and Partial Least Squares (PLS) coefficients are provided in Table 3. The expression of 89 unique genes was visualized on the heatmap with Euclidean clustering and on the Principal Component Analysis (PCA) plot (Figure 4).

The Receiver Operating Characteristics (ROC) analysis was performed to further evaluate the discriminative ability of 89 unique genes. The obtained areas under ROC curves ranged between 1 and 0.939 for analyzed genes, indicating good performance for distinguishing of studied diseases (Table 3). The detailed results of ROC analysis are provided in Table S1.

**Figure 5:**
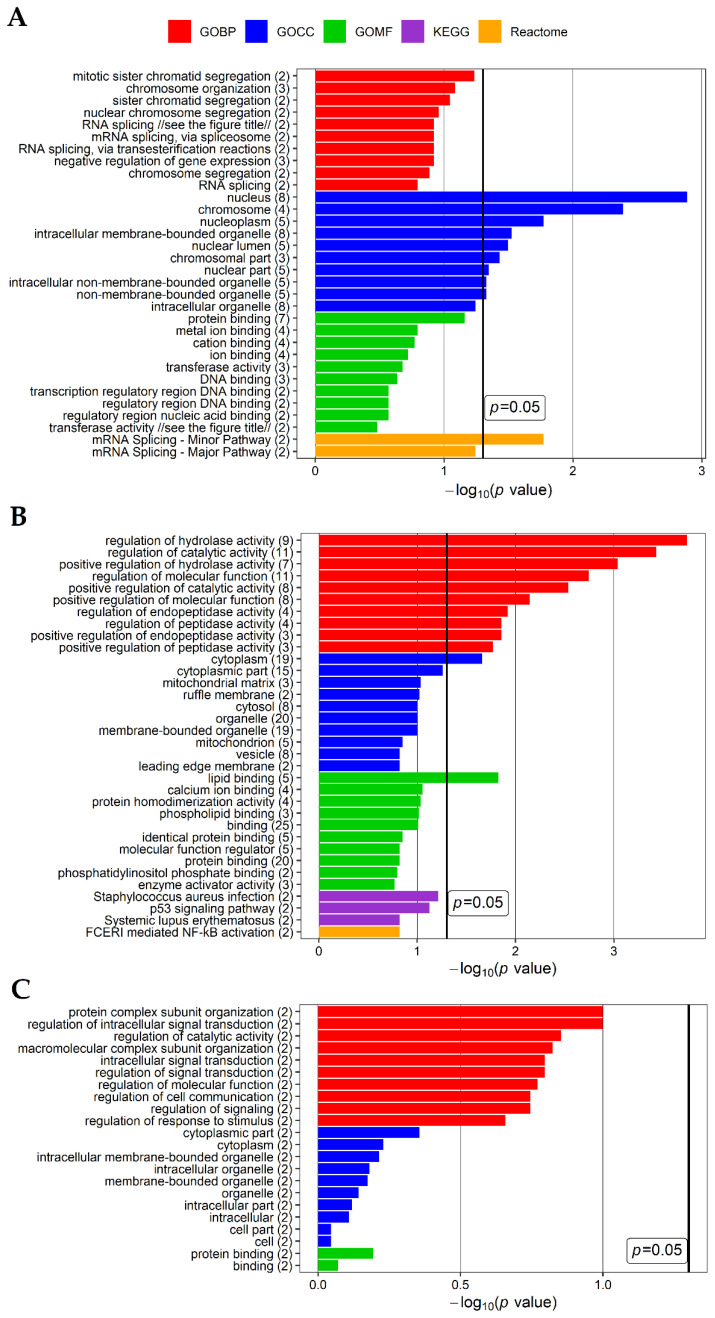
Results of functional analysis performed using DAVID website tool for the sets of (**A**) 21 unique genes for LEAD vs. AAA comparison, (**B**) 58 unique genes for LEAD vs. CVD comparison and (**C**) 10 unique genes for AAA vs. CVD comparison. Similar to Figure 2, up to ten of the most enriched terms of Gene Ontology Biological Processing (GOBP), Gene Ontology Cellular Compartment (GOCC), Gene Ontology Molecular Function (GOMF), KEGG (Kyoto Encyclopedia of Genes and Genomes) and Reactome categories were presented. *p* value—EASE score for enrichment, the black vertical line represents *p* = 0.05 threshold. The number in brackets following the name of terms indicates the number of associated genes. Due to a large length of some names of Gene Ontology terms on panel A, to make this figure clearer, they were shown in shortened form: the full name of “RNA splicing” term is “RNA splicing, via transesterification reactions with bulged adenosine as nucleophile”, the full name of “transferase activity” term is “transferase activity, transferring phosphorus-containing groups”. LEAD—lower extremities arterial disease, AAA—abdominal aortic aneurysm, CVD—chronic venous disease.

**Figure 6:**
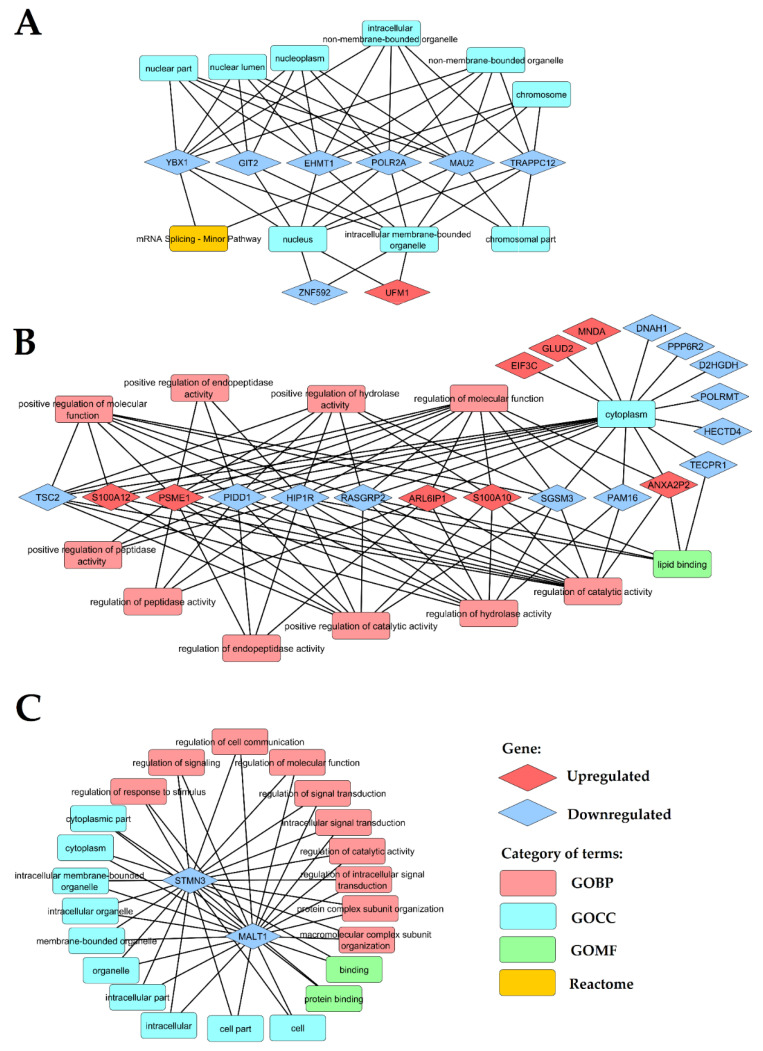
Networks of enriched functional terms and associated genes. The network was constructed using significantly (*p* < 0.05) enriched terms revealed for 21 unique genes selected from LEAD vs. AAA comparison (**A**) and 58 unique genes selected from LEAD vs. CVD comparison (**B**) (refer to Figure 5). Due to the lack of significantly enriched terms for 10 unique genes selected from AAA vs. CVD comparison, all terms presented on Figure 5 were networked (**C**). GOBP—Gene Ontology Biological Processing, GOCC—Gene Ontology Cellular Compartment, GOMF—Gene Ontology Molecular Function, AAA—abdominal aortic aneurysm, CVD—chronic venous disease LEAD—lower extremities arterial disease.

To explore the biological role of unique genes, a set of 21 unique genes selected from the comparison LEAD vs. AAA, 58 unique genes selected from the comparison LEAD vs. CVD and 10 unique genes selected from the comparison AAA vs. CVD were submitted to functional analysis performed by DAVID website tool. Up to top ten the most enriched functional terms for each gene set were harvested and presented on Figure 5. The most enriched terms for 21 genes differentiating LEAD and AAA groups were mainly associated with regulation of posttranscriptional modifications of RNA and translation, the most enriched terms for 58 genes differentiating LEAD and CVD were associated mainly with protein metabolism, and the most enriched terms for 10 genes differentiating AAA and CVD were mainly associated with intracellular signal transduction (Figure 5). Network of functional terms and associated genes is presented on Figure 6.

### 2.4. Identification of Relationships between the Study Group Characteristics and Expression of Genes Found as Unique for LEAD vs. AAA, LEAD vs. CVD and AAA vs. CVD Comparisons

The identified transcriptomic differences between studied diseases could be an effect of not only disease status, but also clinical and demographical characteristics for each studied group. Therefore, the relationships between expression of 89 genes (21 + 58 + 10) identified as unique for LEAD vs. AAA, LEAD vs. CVD and AAA vs. CVD comparisons and characteristics differentiating LEAD, AAA and CVD groups with statistical significance were evaluated. The analyzed characteristics include age, body mass index, smoking and hypertension status, creatinine level, medication with statins, acetylsalicylic acid and beta-adrenergic blockers (Table 1). Analysis of continuous variables (age, body mass index, creatinine level) was performed using Spearman rank correlation test with application of corrected statistical significance *p* < 0.05 and the absolute value of correlation coefficient R ≥ 0.6 as a cutoff threshold. For categorical variables (never and former smokers vs. current smokers, hypertension status, medication with statins, acetylsalicylic acid and beta-adrenergic blockers), a two-sided Mann–Whitney *U* test with corrected *p* < 0.05 as a cutoff threshold was applied.

Among 21 genes unique for LEAD vs. AAA comparison, three were negatively correlated with creatinine level. In the group of 58 genes unique for LEAD vs. CVD comparison, 15 were correlated with age, eight were correlated with BMI and 12 were positively correlated with creatinine level. In the case of 10 genes selected from AAA vs. CVD comparison, four were negatively correlated with age (Table 3). Obtained results show relation of either age, BMI or creatinine level with genes differentiating LEAD and CVD groups, suggesting difference in influence of these characteristics on LEAD and CVD onsets. Obtained correlations could be also a reflective for differences in these characteristics between LEAD and CVD groups, because LEAD group included significantly older subjects with higher BMI and creatinine levels than CVD group (Table 1).

Fifteen genes unique for LEAD and CVD comparison as well as 4 genes unique for AAA vs. CVD comparison were correlated with age (Table 3), what could be result in significant differences in age between compared groups. Interestingly, there were no genes unique from AAA vs. CVD comparison which were correlated with BMI, despite significant differences in BMI between these groups, what may suggest lower impact of these characteristic an AAA than on LEAD development.

In the case of correlation between gene expression and creatinine level, genes downregulated in LEAD vs. AAA were negatively correlated with creatinine levels as well as genes upregulated in LEAD vs. CVD were positively correlated with creatinine levels (Table 2 and Table 3), what may reflect a relationship between the higher creatinine levels in LEAD patients (Table 1) and change in expression of correlated genes. Results of correlation analysis show that differences in gene expression patterns found between compared diseases are at least partially affected by differences in age, BMI and creatinine levels and further investigations of these relationships should be carried out.

The statistically significant relationships between expression of 89 genes identified as unique for LEAD vs. AAA, LEAD vs. CVD and AAA vs. CVD comparisons and categorical characteristics of all patients (smoking and hypertension status, medication with statins, acetylsalicylic acid and beta-adrenergic blockers) are presented in Table 4. None of analyzed genes was found to be related to smoking status, which could be a result of similar proportions of patients who never and former smoked to patients who are current smokers (Table 1). There were also none of analyzed genes which were found to have statistically significant different expression between subjects with and without beta-adrenergic blockers medication. Two genes each from LEAD vs. CVD comparison were found to be linked to hypertension status (*GLI4* and *MNDA*) and usage of statins (*FAM167A* and *C1orf216*) as well as 58 genes (54 and 4 from LEAD vs. CVD and AAA vs. CVD comparisons, respectively) were related to acetylsalicylic acid medication (Table 4). These genes mainly belong to genes differentiating LEAD and CVD groups, characterized by prominent differences in hypertension status and usage of statins and acetylsalicylic acid (Table 1), what could be an substantial factor altering expression of related genes.

Obtained results indicate that hypertension and medication in studied groups could affect transcriptomic profiles of PBMCs and the differential character of analyzed genes could be a result of applied pharmacotherapy, however further studies are needed to evaluate this effect.

## 3. Discussion

High prevalence and the burden of severe complications make LEAD, AAA and CVD together the major health problem worldwide. Often asymptomatic course of disease progress as well as atypical symptoms cause these diseases to be constantly underdiagnosed what creates the need for new tools for detection and assessment. Determination of biological markers using high-accessible biological material, including PBMCs and other blood components, is beneficial for clinical practice. Therefore, in our previous works we used PBMCs to identify dysregulations in miRNA:gene regulatory network in patients with LEAD, AAA and CVD in relation to healthy controls and potential biomarkers were proposed [37,42,46]. In the current paper, transcriptomic differences in PBMCs between studied diseases were investigated and potential implications to pathogenesis were explored.

In the first approach, upregulation of *GGT1* was identified as a common marker of LEAD, AAA and CVD subjects after comparison to healthy controls (Figure 1). This finding is in concordance with previous studies, where increased serum level of GGT was found to be associated with peripheral arterial disease (PAD) in non-alcoholic males [47] and with higher risk of subclinical coronary atherosclerosis, coronary artery calcification and cardiac events [48,49]. Elevated serum GGT levels are related to increased concentrations of homocysteine [50], which is well known independent risk factor of cardiovascular diseases, including LEAD [51]. Upregulation of *GGT1* was also found in aortic tissue wall of AAA patients [52]. Increased GGT level is considered as an adaptation to higher oxidative stress, since GGT plays an important role in glutathione homeostasis by providing cysteine for intracellular *de novo* synthesis of glutathione via breaking down extracellularly localized glutathione [53]. GGT is also involved in arachidonic acid metabolism through catalyzing of leukotriene D_4_ (LTD4) formation [54], a factor stimulating vascular inflammation [55]. Both *GGT1* and LTD4 circulatory levels were demonstrated to be elevated in individuals exposed to acute hypoxia [56]. These finding suggests that the upregulation of *GGT1* in LEAD and AAA could be a hallmark of oxidative stress, inflammation and hypoxia, which are pertinent elements of these diseases’ onsets.

In the second approach, differential gene expression analysis was performed using DESeq2 and UVE-PLS methods for the following comparisons: LEAD vs. AAA, LEAD vs. CVD and AAA vs. CVD. From each comparison, genes meeting unified cutoff criteria (*p* value adjusted by Benjamini–Hochberg false discovery rate <0.001 for DESeq2 results and reliability score ≥8 for UVE-PLS results) were selected. 21, 58 and 10 genes were selected from LEAD vs. AAA, LEAD vs. CVD and AAA vs. CVD, respectively (Figure 3, Table 2). Interestingly, there were no genes overlapping in all three sets (Figure 3D), therefore selected genes could be considered as specific for corresponding comparisons and may carry abundant information about differences in transcriptomic patterns of studied diseases.

Differentially expressed genes identified as specific for performed comparisons formed a transcriptional landscape of every disease, pointing out differences between them. The striking observation was a clear bias towards enrichment in genes of various regulatory potential, such as pseudogenes (Table 5). Due to advancement of high-throughput sequencing platforms, it was shown, that a high number of pseudogenes is indeed transcriptionally active and could be functionally significant part of the genome [57,58,59].

Pseudogene regulatory function may be elucidated by many different mechanisms such as being a microRNA sponge, recruiting chromatin-remodeling factors to its parental gene, acting as a decoy for RNA-binding proteins, associating with DNA-binding transcription factors and interrupting the DNA binding capacity [60].

Upregulation of genes belong to pseudogene class is a prominent feature distinguishing LEAD from CVD, comprising 76.7% (Table 5) of all upregulated specific genes. This may reveal potential complex mode of regulation in LEAD as a subtype of atherosclerosis and may reflect relative richness of atherosclerotic symptoms and characteristics.

The majority of genes with lower expression in AAA group in relation to LEAD and CVD patients belongs to small nucleolar RNAs (snoRNAs) of C/D box and H/ACA box types (Table 5). The function snoRNAs is 2′-O-ribose methylation and pseudouridylation of ribosomal RNAs [61], which suggests different regulation of translation process in AAA when compared to LEAD and CVD.

*SNHG5* was reported as a pro-oncogenic factor stimulating proliferation of myeloid leukemia cells [62]. High expression of two snoRNAs, *SNORA72* and *SNORA26*, was correlated with pro-oncogenic properties of cells and poor prognosis in cancer [63,64]. Upregulation of these genes in LEAD vs. AAA subjects may promote proliferation of immune cells and reflect higher inflammation status of the atherosclerotic lesions compared to aneurysm.

Four out of 12 genes downregulated in LEAD vs. AAA patients belong to *RN7SK* miscellaneous RNAs (*RN7SKP7*, *RN7SKP45*, *RN7SKP208*, *RN7SKP286*) (Table 5). For the time being, there were no reports about contribution of those genes to cardiovascular diseases or any biological process (according to GWAS Catalog database [65], accessed 15 January 2021). Despite lack of data, their function could be elucidated on the known functions of the *RN7SK* gene. Its product is a long, non-coding RNA which contributes to control of transcription elongation by RNA polymerase II [66,67]. One can speculate, that miscellaneous *RN7SK* RNAs found in our study may coregulate the AAA transcriptome in a global manner.

Upregulation of *UFM1* was demonstrated in LEAD vs. AAA group (Table 2). *UFM1* encodes evolutionarily conserved ubiquitin-like protein triggering activation of protein targets through UFMylation. UFMylation process is essential for erythropoiesis by maintaining proper survival and differentiation of cells in erythroid lineage. Knockout of UFMylation pathway downstream effectors in animals causes severe anemia associated with defective differentiation of both megakaryocytes and erythrocytes [68]. Higher expression of *UFM1* in LEAD patients when compared to AAA subjects may reflect enhanced erythropoiesis stimulated by chronic ischemia characteristic for LEAD progression.

The presented study show downregulation of *EHMT1* in LEAD vs. AAA group (Table 2). Lower expression level of *EHMT1* was shown to be a factor increasing fetal hemoglobin levels [69], which is positively correlated with carotid artery intima media thickness in patients with β-thalassemia major [70]. The influence of atherosclerosis status of fetal hemoglobin levels seems to be an interesting topic for future studies on biomarkers of atherosclerosis. Moreover, *EHMT1* together with *EHMT2* regulate alternative splicing of *VEGFA* [71], which antiangiogenic splice isoform VEGF-A165b was shown to be elevated in serum of LEAD patients with coexistence of the reduction of the proangiogenic VEGF-A165a isoform [72]. The possible influence of *EHMT1* dysregulation on biosynthesis of these VEGFA splicing isoforms in the context of vascular diseases should be further investigated.

Downregulation of *YBX1* observed in the current study in LEAD vs. AAA subjects (Table 2) may indicate enhanced oxidized LDL-mediated inflammatory response and lipid deposition in macrophages [73], what is characteristic for pathogenesis of atherosclerosis, and hence LEAD.

*GIT2* was identified as a key regulator of complex ageing processes, including DNA damage, oxidative stress, metabolic disruption, inflammation and fat deposition. *GIT2* promote DNA repair and its age-dependent increase reflects a cellular protection mechanism attenuating ROS-induced DNA damage. Elevation in *GIT2* expression levels was reported in an animal model for diabetes and obesity. *GIT2* is also involved in regulation of inflammaging, which is an age-related, progressive increase in low-grade chronic inflammation linked to elevated levels of inflammatory biomarkers such as C-reactive protein and interleukin IL-6 [74]. Higher expression of *GIT2* in AAA patients in relation to LEAD subjects (Table 2) suggests enhanced association of aging hallmarks with AAA pathogenesis in comparison to LEAD, what is especially suggestive, because individuals in the AAA and LEAD groups have similar age.

*S100A10* and *S100A2* were shown in presented research as to be upregulated in LEAD vs. CVD group (Table 2). *S100A10* was previously shown to be hypomethylated in coronary artery plaques compared to great saphenous vein [75], what may be an explanation of higher expression of this gene in LEAD group. Higher expression of *S100A12* was identified as a biomarker of coronary artery disease, aortic calcification, increased plaque vulnerability and as predictor of cardiovascular events [76]. Both genes were identified as being involved in macrophage activation and inflammatory cytokines induction [75,76]. Implications of *S100A10* and *S100A12* in atherosclerosis could entail differences in their expression in LEAD and CVD groups, however acetylic acid medication could also be causative for obtained results (Table 5).

Upregulation of *SGSM3* in CVD vs. LEAD group (Table 2) may reflect higher ROS status in CVD, due to protective role of *SGSM3* against oxidative stress [77].

In CVD group, *TSC2* exhibits higher expression when compared to LEAD group (Table 2). It may suggest less intense cellular proliferation in CVD since *TSC2* was evidenced to exert suppressing effect on cell cycle through inhibition of mTOR signaling [78]. Lower expression of *TSC2* in LEAD may be an effect of aging, a process with declined AMP-activated protein kinase (AMPK) signaling, which activates *TSC2* [78]. One of the features of aging related to suppression of AMPK is lowering the autophagy, a process essential for degradation of protein aggregates forming in aging cells [79]. Lower level of autophagy could be also indicated by downregulation of promotor of aggrephagy *TCPR1* in LEAD group [80]. These findings could reflect a more advanced aging process in LEAD vs. CVD group, but may also be a result of significant difference in age between these groups.

Another gene downregulated in LEAD after comparison to CVD group is *RASGRP2* (Table 2), which promotes adhesion of T cells and contribute to the endothelial homeostasis via preventing TNF-induced ROS production and apoptosis in umbilical vein endothelial cells (HUVECs) [81]. Higher expression of *RASGRP2* in CVD vs. LEAD may be a stimulus for venous inflammation and lower expression in LEAD could be a hallmark of endothelium dysfunction, however further studies on this topic are required.

*MIR150* is a gene downregulated in AAA vs. CVD and also negatively correlated with age and potentially linked to acetylsalicylic acid usage (Table 2, Table 3 and Table 4). This gene encodes mature miRNA miR-150-5p, whose downregulation was reported in patients with AAA and subaneurysmal aortic dilation in relation to high cardiovascular risk subjects with normal aortic diameter [82]. Aberrant expression of this miRNA seems to be triggered by disease status rather than by age, because age-matched populations were investigated in [82]. Lower expression of miR-150-5p may indicate AAA-associated endothelial dysfunction and vascular remodeling, since this miRNA has been suggested to play protective role by maintaining endothelium function and suppressing vascular remodeling via inhibition of pentaxin-3 expression [83].

Another gene with expression higher in CVD when compared to AAA is *MALT1* (Table 2), which is a component of CARD11–BCL10–MALT1 signalosome, triggering inflammatory pathways in activated leukocytes and mediate lymphocyte proliferation, differentiation, metabolic reprogramming and survival after antigen recognition. The CARD10–BCL-10–MALT1 complex is a regulator of cardiovascular inflammation and remodeling though induction of cytokine and chemokine production in either endothelial or vascular smooth muscle cells. Moreover, this complex mediates the disruption of the endothelial barrier [84]. Higher expression of *MALT1* in CVD patients may be a hallmark of vascular inflammation and remodeling ongoing in vein tissues during CVD.

Three genes (*POLR2A*, *ZNF592*, *TRAPPC12*) differentiating LEAD from AAA and 12 genes (*RP11-262D11.2*, *SRRM1P3*, *SDCBPP2*, *ARL6IP1*, *HNRNPA1P7*, *API5P1*, *AC104651.2*, *EIF3FP3*, *RP11-286H14.4*, *CTNNA1P1*, *DYNC1I2P1*, *CTB-52I2.4*) differentiating LEAD and CVD were also correlated with creatinine levels (Table 3). Altered expression of these genes probably reflect higher levels of creatinine observed in LEAD patients (Table 1). High creatinine level is a hallmark of declined glomerular filtration rate, a typical clinical marker of kidney failure. Chronic kidney disease was previously reported to be strongly associated with LEAD and other markers of kidney function such as cystatin C and β2-microglobulin were shown to be better markers of LEAD risk in patients with kidney diseases [85,86,87]. *POLR2A* gene, which is shown in the current study as downregulated in LEAD vs. AAA (Table 2) and negatively correlated with creatinine level (Table 3), was previously found as involved in congenital obstructive nephropathy [88] and encoded protein was strongly functionally linked to dysregulated proteins in animal models of salt-induced kidney damage [89]. The implications of *POLR2A* and other dysregulated genes correlated with creatinine levels in cross-talks between LEAD and kidney failure onsets should be a subject of further studies.

A descriptive character of our study raised some limitations. Application of unified cutoff criteria for selection of differentially expressed genes, although increasing the comparability of obtained results, could cause loss of certain genes important for differentiating diseases studied. Due to statistically significant differences in demographic and clinical characteristics of compared groups, selected differentially expressed genes could be not exclusive for disease status, but their expression levels could be affected by other analyzed variables, especially age, BMI, creatinine levels, hypertension and medication. Moreover, changes in lymphocytes and monocytes subpopulations content in PBMCs samples, however assessed as not significant (Figure S5), could be a potential source of bias in obtained transcriptome profiles.

To recompensate the lack of qPCR validation, we decided to use broader and more advance statistical analysis (DESeq2 with UVE-PLS confirmation, ROC analysis) and took much stricter thresholds of statistical significance (*p* < 0.001) with Benjamini–Hochberg false discovery rate correction into account in order to substantially limit potential false positive results.

Finally, due to initial and descriptive character of the presented study, the conclusions inferred from obtained results need to be confirmed in explanatory studies. Differential character of determined gene signatures and their role in vascular pathology should be elucidated in detail in further studies using such techniques as qPCR, flow cytometry, western blot, transfection methods or experiments with animal models. Further validation studies should also include investigations in much larger and more balanced populations.

The aim of sharing findings of the current work was to give the opportunity to start a discussion within scientific community and to propose new explorative paths for other research groups.

## 4. Materials and Methods

### 4.1. Study Participants

The study was performed in accordance with the Declaration of Helsinki and after approval of the Bioethics Commission of the Medical University of Lublin (decision No. KE-0254/341/2015, approval date 17 December 2015). The study group consists of 8 patients with LEAD, 7 patients with AAA and 7 patients with CVD. All individuals were diagnosed in Independent Public Clinical Hospital No. 1 in Lublin between February 2016 and May 2017. Informed and signed consent was obtained from all study subjects. Detailed inclusion procedure and established exclusion criteria were provided in our previous papers [37,42,46]. Clinical characteristics of participants are presented in Table 1 and detailed clinical features specific for each disease was provided in Table A1 in Appendix A.

### 4.2. Gene Expression Datasets

Gene expression datasets were generated by RNA sequencing of PBMCs samples obtained from the study participants as described in our previous papers. Briefly, PBMCs specimens were isolated from whole blood samples using density gradient centrifugation with Gradisol L reagent (Aqua-Med, Łódź, Poland). A diversity of white blood cells subpopulations in studied groups were evaluated using the whole blood morphology analysis (Figure S5). Total RNA was isolated from PBMCs samples using TRI Reagent Solution (Applied Biosystems, Foster City, CA, USA). Total RNA samples underwent ribodepletion procedure using RiboMinus Eukaryote System v2 (Ambion, Austin, TX, USA) and were subjected to whole transcriptome libraries preparation using Ion Total RNA-Seq Kit v2, Magnetic Bead Cleanup Module kit and Ion Xpress RNA-Seq Barcode 01-16 Kit (Life Technologies, Carlsbad, CA, USA). Libraries were sequenced on Ion 540 chips (Life Technologies) using Ion S5 XL System (Thermo Fisher Scientific, Waltham, MA, USA). Raw sequences were aligned to 55,765 genes of hg19 human genome using Torrent Suite Software v5.0.4. and Ion Torrent RNASeqAnalysis plugin v.5.0.3.0 (Thermo Fisher Scientific). Statistics of parameters describing transcriptome libraries and primary results of sequencing data analysis are provided in Table S2.

### 4.3. Data Analysis

Data analysis was performed using R environment (version 3.6.3, https://www.r-project.org, accessed on 12 January 2021) and appropriate packages according to corresponding reference manuals.

Statistical significance of differences in demographic and clinical parameters between LEAD, AAA and CVD groups was examined using Kruskal-Wallis rank sum test for continuous variables (kruskal.test function in R) and two-sided Fisher’s exact test for categorical variables (fisher.test function in R).

All further statistical procedures applied to expression datasets and subsequent bioinformatical analysis were previously described in detail in [37,42,46].

Differential expression analysis of whole transcriptome expression datasets was performed on biological replicates using DESeq2 method implemented in DESeq2 1.26.0 package [90] (https://bioconductor.org/packages/release/bioc/html/DESeq2.html, accessed on 12 January 2021) and Uninformative Variable Elimination by Partial Least Squares (UVE-PLS) method [91] implemented in plsVarSel 0.9.6 package [92] (https://cran.r-project.org/web/packages/plsVarSel/index.html, accessed on 12 January 2021).

DESeq2 analysis was performed on expression data filtered out of genes with mean of reads lower than one. Differentially expressed genes received from DESeq2 analysis with *p* value below 0.001 after Benjamini–Hochberg false discovery rate correction were considered as statistically significant.

For UVE-PLS analysis, filtered expression data was transformed using regularized log normalization (rlog function in DESeq2 package). UVE-PLS analysis was performed with 1000 iterations, reliability score cutoff threshold equal to 8 and 0.75 ratio for splitting into trained and tested data subsets. An optimal number of PLS (Partial Least Squares) components for UVE-PLS analysis (Table A2 in Appendix B) was established by PLS regression with leave-one-out (LOO) cross-validation followed by visual inspection of plots presenting the arrangement of estimated Root Mean Squared Error of Prediction (RMSEP) over the number of PLS components (Figure S6).

Venn diagrams, heatmap with Euclidean clustering and PCA plot were created using VennDiagram 1.6.20 (https://cran.r-project.org/web/packages/VennDiagram/index.html, accessed on 15 January 2021) [93], pheatmap 1.0.12 (https://cran.r-project.org/web/packages/pheatmap/index.html, accessed on 15 January 2021) and ggplot2 3.3.0 (https://ggplot2.tidyverse.org, accessed on 15 January 2021) packages, respectively.

A ROC analysis implemented in pROC package 1.16.2 [94] (https://cran.r-project.org/web/packages/pROC/index.html, accessed on 16 January 2021) was used to evaluate the predictive value of selected genes.

Identification of relationships between characteristics of study participants and expression of selected genes were performed using Spearman rank correlation test implemented in Hmisc package 4.4-0. (https://cran.r-project.org/web/packages/Hmisc/index.html, accessed on 18 January 2021) as well as a two-sided Mann–Whitney *U* test implemented in wilcox.test function in R.

Functional analysis of selected genes was performed using Database for Annotation, Visualization and Integrated Discovery (DAVID) 6.8 tool (https://david.ncifcrf.gov/, accessed on 21 January 2021) [95,96]. Default whole genome of *Homo sapiens* was applied as a background. Up to ten top the most enriched terms were selected from Gene Ontology, KEGG and Reactome categories. Functional network of the most enriched terms and associated genes was constructed using Cytoscape v3.7.0 software (https://cytoscape.org/, accessed on 23 January 2021) [97].

## 5. Conclusions

In our work we demonstrated that PBMCs are a valuable material to investigate transcriptomic differences between patients with LEAD, AAA and CVD. Analysis of PBMCs gene expression profiles enabled finding of associations of selected genes with characteristics of participants and their implications in disease pathogenesis. Performed identification of potentially disease-specific biomarkers could provide new diagnostic and therapeutic opportunities in LEAD, AAA and CVD management; however, further validation in studies on larger and demographically matched populations is required to launch implementation process of our results to clinical practice.

## Figures and Tables

**Table 1:** Clinical characteristics of the study subjects.

Characteristic	LEAD (*n* = 8)	AAA (*n* = 7)	CVD (*n* = 7)	*p* ^1^
Age	62 ± 7.82 ^2^	66.3 ± 4.03 ^2^	41.3 ± 4.03 ^2^	8.273 × 10^−4 4^
	48–71 ^3^	59–71 ^3^	35–47 ^3^	
Gender males/females	6 (75%)/2 (25%)	6 (85.7%)/	3 (42.9%)/	0.275 ^5^
		1 (14.3%)	4 (57.1%)	
Body mass index (BMI)	28.25 ± 2.07 ^2^	27.23 ± 2.76 ^2^	23.36 ± 1.94 ^2^	6.272 × 10^−3 4^
	25.5–31.2 ^3^	23.66–30.85 ^3^	20.94–25.83 ^3^	
**Risk factors and cardiovascular comorbidities**				
Smoking never/former/current	0 (0%)/6 (75%)/	3 (42.9%)/	5 (71.4%)/0 (0%)/	0.011 ^5^
	2 (25%)	2 (28.6%)/2 (28.6%)	2 (28.6%)	
Diabetes type 2	3 (37.5%)	2 (28.6%)	0 (0%)	0.300 ^5^
Hypertension	7 (87.5%)	5 (71.4%)	0 (0%)	9.418 × 10^−4 5^
Coronary artery disease (CAD)	2 (25%)	1 (14.3%)	0 (0%)	0.746 ^5^
Myocardial infarction	2 (25%)	1 (14.3%)	0 (0%)	0.746 ^5^
Stroke/Transient ischemic attack	0 (0%)	0 (0%)	0 (0%)	1.000 ^5^
**Hematological and biochemical blood parameters**				
Red blood cells (M/µl)	4.81 ± 0.33 ^2^	4.96 ± 0.19 ^2^	4.93 ± 0.31 ^2^	0.630 ^4^
	4.22–5.18 ^3^	4.56–5.10 ^3^	4.29–5.21 ^3^	
White blood cells (K/µl)	5.49 ± 0.69 ^2^	5.85 ± 0.75 ^2^	5.58 ± 0.50 ^2^	0.677 ^4^
	4.79–6.70 ^3^	4.89–6.89 ^3^	4.67–5.99 ^3^	
Platelets (K/µl)	348.5 ± 105.5 ^2^	379.43 ± 82.26 ^2^	368.14 ± 66.26 ^2^	0.430 ^4^
	267–432 ^3^	267–501 ^3^	295–467 ^3^	
Hemoglobin (g/dl)	14.22 ± 0.59 ^2^	13.88 ± 0.52 ^2^	13.98 ± 0.33 ^2^	0.415 ^4^
	13.45–14.80 ^3^	13.34–14.60 ^3^	13.56–14.60 ^3^	
Hematocrit (%)	40.91 ± 1.15 ^2^	41.31 ± 1.13 ^2^	40.24 ± 2.35 ^2^	0.425 ^4^
	38.9–42 ^3^	39.9–43 ^3^	37.00–44 ^3^	
Creatinine (mmol/L)	80.38 ± 11.11 ^2^	58.86 ± 11.60 ^2^	58.71 ± 8.75 ^2^	4.529 × 10^−3 4^
	59–89 ^3^	44–77 ^3^	45–67 3	
Urea (mmol/L)	4.69 ± 0.70 ^2^	4.61 ± 0.47 ^2^	4.77 ± 0.98 ^2^	0.931 ^4^
	3.70–6.01 ^3^	3.89–5.10 ^3^	3.78–6.37 ^3^	
**Medication**				
Statins	7 (87.5%)	4 (57.1%)	0 (0%)	2.818 × 10^−3 5^
Acetylsalicylic acid	8 (100%)	7 (100%)	0 (0%)	1.173 × 10^−5 5^
Clopidogrel	2 (25%)	0 (0%)	0 (0%)	0.303 ^5^
Beta-adrenergic blockers	6 (75%)	5 (71.4%)	0 (0%)	8.375 × 10^−3 5^
Angiotensin-converting enzyme inhibitor	2 (25%)	0 (0%)	0 (0%)	0.303 ^5^
Ca^2+^ channel blockers	3 (37.5%)	1 (14.3%)	0 (0%)	0.270 ^5^
Fibrates	3 (37.5%)	1 (14.3%)	0 (0%)	0.270 ^5^
Metformin	1 (12.5%)	0 (0%)	0 (0%)	1.000 ^5^
Gliclazide	3 (37.5%)	2 (28.6%)	0 (0%)	0.300 ^5^

^1^ Statistical significance of differences between lower extremities arterial disease (LEAD), abdominal aortic aneurysm (AAA) and chronic venous disease (CVD) groups, ^2^ mean ± SD, ^3^ range, ^4^ *p* value calculated using Kruskal-Wallis rank sum test, ^5^ *p* value calculated using two-sided Fisher’s exact test.

**Table 2:** Differential expression parameters of 21, 58 and 10 genes identified as unique for LEAD vs. AAA, LEAD vs. CVD and AAA vs. CVD comparisons, respectively. The table presents *p* (FDR with Benjamini–Hochberg correction) and fold change values received from DESeq2 analysis, PLS coefficients received from UVE-PLS analysis and areas under ROC curves (ROC-AUC) received from ROC analysis. Genes were divided into upregulated and downregulated groups within each comparison and ordered according to increasing *p* value. Gene symbols without assigned gene names by HUGO Gene Nomenclature Committee Multi-symbol checker (https://www.genenames.org/tools/multi-symbol-checker/, accessed on 20 January 2021) were named as “Unmatched”.

No.	Gene Symbol	Gene Name	*p*	Fold Change	PLS Coefficient	ROC-AUC
**LEAD vs. AAA—Upregulated Genes**						
1.	*SNORD20*	small nucleolar RNA, C/D box 20	1.712 × 10^−7^	3.338	2.746 × 10^−3^	1.000
2.	*SNORA72*	small nucleolar RNA, H/ACA box 72	1.205 × 10^−4^	2.103	1.551 × 10^−3^	0.964
3.	*SNHG5*	small nucleolar RNA host gene 5	1.205 × 10^−4^	1.984	1.541 × 10^−3^	1.000
4.	*SNORA26*	small nucleolar RNA, H/ACA box 26	1.358 × 10^−4^	2.365	1.857 × 10^−3^	1.000
5.	*SNORD82*	small nucleolar RNA, C/D box 82	1.489 × 10^−4^	2.206	1.721 × 10^−3^	1.000
6.	*UFM1*	ubiquitin fold modifier 1	2.157 × 10^−4^	1.391	7.038 × 10^−4^	1.000
7.	*SNORD101*	small nucleolar RNA, C/D box 101	3.292 × 10^−4^	2.352	1.757 × 10^−3^	0.964
8.	*SNORD91B*	small nucleolar RNA, C/D box 91B	6.538 × 10^−4^	2.493	1.987 × 10^−3^	1.000
9.	*SNORD111B*	small nucleolar RNA, C/D box 111B	7.373 × 10^−4^	2.337	1.910 × 10^−3^	1.000
**LEAD vs. AAA—downregulated genes**						
10.	*POLR2A*	RNA polymerase II subunit A	2.583 × 10^−5^	0.764	−6.306 × 10^−4^	1.000
11.	*AC092620.2*	Unmatched	2.583 × 10^−5^	0.401	−1.694 × 10^−3^	1.000
12.	*EHMT1*	euchromatic histone lysine methyltransferase 1	2.854 × 10^−5^	0.744	−6.428 × 10^−4^	1.000
13.	*TRAPPC12*	trafficking protein particle complex 12	1.313 × 10^−4^	0.762	−5.972 × 10^−4^	0.980
14.	*RN7SKP286*	RN7SK pseudogene 286	1.313 × 10^−4^	0.143	−3.159 × 10^−3^	0.964
15.	*ZNF592*	zinc finger protein 592	5.389 × 10^−4^	0.740	−6.769 × 10^−4^	1.000
16.	*YBX1*	Y-box binding protein 1	5.525 × 10^−4^	0.625	−9.797 × 10^−4^	0.982
17.	*RN7SKP208*	RN7SK pseudogene 208	5.525 × 10^−4^	0.292	−1.571 × 10^−3^	0.982
18.	*RN7SKP45*	RN7SK pseudogene 45	5.525 × 10^−4^	0.213	−2.861 × 10^−3^	0.982
19.	*RN7SKP7*	RN7SK pseudogene 7	5.525 × 10^−4^	0.199	−1.277 × 10^−3^	1.000
20.	*MAU2*	MAU2 sister chromatid cohesion factor	6.538 × 10^−4^	0.804	−4.892 × 10^−4^	0.982
21.	*GIT2*	GIT ArfGAP 2	9.198 × 10^−4^	0.768	−5.651 × 10^−4^	1.000
**LEAD vs. CVD—upregulated genes**						
1.	*CALM2P2*	calmodulin 2 pseudogene 2	4.927 × 10^−6^	2.622	1.572 × 10^−3^	1.000
2.	*RP11-490H24.5*	Unmatched	9.430 × 10^−6^	3.231	1.296 × 10^−3^	1.000
3.	*RP11-334L9.1*	Unmatched	1.438 × 10^−5^	3.236	1.468 × 10^−3^	0.982
4.	*API5P1*	apoptosis inhibitor 5 pseudogene 1	3.627 × 10^−5^	2.592	1.284 × 10^−3^	1.000
5.	*PDIA3P1*	protein disulfide isomerase family A member 3 pseudogene 1	3.627 × 10^−5^	1.968	1.090 × 10^−3^	1.000
6.	*ARL6IP1*	ADP ribosylation factor like GTPase 6 interacting protein 1	3.627 × 10^−5^	1.540	8.133 × 10^−4^	1.000
7.	*RP11-1033A18.1*	Unmatched	4.570 × 10^−5^	2.266	1.376 × 10^−3^	1.000
8.	*EIF4A1P10*	eukaryotic translation initiation factor 4A1 pseudogene 10	5.014 × 10^−5^	2.026	1.131 × 10^−3^	1.000
9.	*RP11-262D11.2*	Unmatched	5.014 × 10^−5^	1.913	1.072 × 10^−3^	0.946
10.	*S100A10*	S100 calcium binding protein A10	5.014 × 10^−5^	1.723	9.939 × 10^−4^	1.000
11.	*CFL1P4*	cofilin 1 pseudogene 4	5.355 × 10^−5^	2.826	1.400 × 10^−3^	1.000
12.	*AC078899.1*	Unmatched	5.355 × 10^−5^	2.411	1.334 × 10^−3^	0.982
13.	*CAP1P2*	CAP1 pseudogene 2	7.322 × 10^−5^	2.104	1.191 × 10^−3^	1.000
14.	*HNRNPA1P7*	heterogeneous nuclear ribonucleoprotein A1 pseudogene 7	7.322 × 10^−5^	1.814	1.017 × 10^−3^	1.000
15.	*FCGR3B*	Fc fragment of IgG receptor IIIb	9.228 × 10^−5^	3.135	1.917 × 10^−3^	1.000
16.	*CTNNA1P1*	catenin alpha 1 pseudogene 1	9.228 × 10^−5^	3.030	1.412 × 10^−3^	0.982
17.	*PSME1*	proteasome activator subunit 1	9.228 × 10^−5^	1.744	1.083 × 10^−3^	1.000
18.	*RP11-6B6.3*	Unmatched	1.126 × 10^−4^	3.206	1.602 × 10^−3^	1.000
19.	*MSNP1*	moesin pseudogene 1	1.602 × 10^−4^	2.059	1.213 × 10^−3^	1.000
20.	*ACTR3P2*	ACTR3 pseudogene 2	1.640 × 10^−4^	2.564	1.369 × 10^−3^	1.000
21.	*RP13-104F24.3*	Unmatched	1.640 × 10^−4^	2.143	8.857 × 10^−4^	0.982
22.	*HSP90B3P*	heat shock protein 90 beta family member 3, pseudogene	1.987 × 10^−4^	2.373	1.277 × 10^−3^	1.000
23.	*DYNC1I2P1*	dynein cytoplasmic 1 intermediate chain 2 pseudogene 1	2.024 × 10^−4^	2.441	1.344 × 10^−3^	1.000
24.	*EIF3FP3*	eukaryotic translation initiation factor 3 subunit F pseudogene 3	2.996 × 10^−4^	1.976	1.059 × 10^−3^	0.964
25.	*C1orf216*	chromosome 1 open reading frame 216	3.042 × 10^−4^	1.474	6.989 × 10^−4^	0.982
26.	*ANXA2P2*	annexin A2 pseudogene 2	3.767 × 10^−4^	2.368	1.258 × 10^−3^	1.000
27.	*MNDA*	myeloid cell nuclear differentiation antigen	4.212 × 10^−4^	2.198	1.322 × 10^−3^	1.000
28.	*AC104651.2*	Unmatched	4.292 × 10^−4^	3.349	9.316 × 10^−4^	0.946
29.	*PGDP1*	phosphogluconate dehydrogenase pseudogene 1	4.292 × 10^−4^	2.653	1.284 × 10^−3^	0.982
30.	*PSME2P2*	proteasome activator subunit 2 pseudogene 2	4.425 × 10^−4^	2.547	1.508 × 10^−3^	1.000
31.	*CDC42P6*	cell division cycle 42 pseudogene 6	4.693 × 10^−4^	1.981	1.051 × 10^−3^	1.000
32.	*HSP90B2P*	heat shock protein 90 beta family member 2, pseudogene	5.142 × 10^−4^	2.048	1.107 × 10^−3^	1.000
33.	*HSPA9P1*	heat shock protein family A (Hsp70) member 9 pseudogene 1	5.302 × 10^−4^	1.930	8.806 × 10^−4^	1.000
34.	*C1QB*	complement C1q B chain	5.647 × 10^−4^	5.492	2.159 × 10^−3^	0.964
35.	*CTB-52I2.4*	Unmatched	5.855 × 10^−4^	2.077	1.013 × 10^−3^	0.982
36.	*RP11-286H14.4*	Unmatched	5.907 × 10^−4^	1.932	9.926 × 10^−4^	1.000
37.	*SETP14*	SET pseudogene 14	6.672 × 10^−4^	1.785	9.681 × 10^−4^	1.000
38.	*CALM2P4*	calmodulin 2 pseudogene 4	6.970 × 10^−4^	2.329	1.066 × 10^−3^	1.000
39.	*GLUD2*	glutamate dehydrogenase 2	7.874 × 10^−4^	1.870	8.908 × 10^−4^	0.982
40.	*EIF3C*	eukaryotic translation initiation factor 3 subunit C	8.845 × 10^−4^	1.670	9.814 × 10^−4^	1.000
41.	*SDCBPP2*	syndecan binding protein pseudogene 2	9.306 × 10^−4^	2.454	1.164 × 10^−3^	1.000
42.	*SRRM1P3*	serine/arginine repetitive matrix 1 pseudogene 3	9.306 × 10^−4^	2.044	1.077 × 10^−3^	1.000
43.	*S100A12*	S100 calcium binding protein A12	9.443 × 10^−4^	2.972	1.516 × 10^−3^	0.946
**LEAD vs. CVD—downregulated genes**						
44.	*TSC2*	TSC complex subunit 2	3.328 × 10^−6^	0.765	−5.314 × 10^−4^	1.000
45.	*SGSM3*	small G protein signaling modulator 3	5.014 × 10^−5^	0.723	−5.758 × 10^−4^	1.000
46.	*TECPR1*	tectonin beta-propeller repeat containing 1	6.319 × 10^−5^	0.716	−6.552 × 10^−4^	1.000
47.	*RASGRP2*	RAS guanyl releasing protein 2	7.322 × 10^−5^	0.663	−7.661 × 10^−4^	0.964
48.	*GLI4*	GLI family zinc finger 4	1.484 × 10^−4^	0.671	−6.852 × 10^−4^	1.000
49.	*PPP6R2*	protein phosphatase 6 regulatory subunit 2	1.640 × 10^−4^	0.773	−5.375 × 10^−4^	1.000
50.	*TBC1D27P*	TBC1 domain family member 27, pseudogene	1.806 × 10^−4^	0.220	−2.347 × 10^−3^	1.000
51.	*D2HGDH*	D-2-hydroxyglutarate dehydrogenase	2.024 × 10^−4^	0.589	−9.321 × 10^−4^	0.964
52.	*DNAH1*	dynein axonemal heavy chain 1	2.532 × 10^−4^	0.727	−5.605 × 10^−4^	1.000
53.	*PAM16*	presequence translocase associated motor 16	3.180 × 10^−4^	0.526	−1.073 × 10^−3^	0.982
54.	*HIP1R*	huntingtin interacting protein 1 related	3.236 × 10^−4^	0.489	−1.198 × 10^−3^	1.000
55.	*FAM167A*	family with sequence similarity 167 member A	4.088 × 10^−4^	0.331	−1.221 × 10^−3^	0.982
56.	*PIDD1*	p53-induced death domain protein 1	4.292 × 10^−4^	0.682	−6.748 × 10^−4^	1.000
57.	*HECTD4*	HECT domain E3 ubiquitin protein ligase 4	5.855 × 10^−4^	0.785	−4.282 × 10^−4^	0.982
58.	*POLRMT*	RNA polymerase mitochondrial	6.453 × 10^−4^	0.710	−5.682 × 10^−4^	1.000
**AAA vs. CVD—downregulated genes**						
1.	*SNORA11*	small nucleolar RNA, H/ACA box 11	2.066 × 10^−6^	0.392	−1.585 × 10^−3^	0.980
2.	*SNORD64*	small nucleolar RNA, C/D box 64	4.692 × 10^−6^	0.354	−1.471 × 10^−3^	0.959
3.	*MIR150*	microRNA 150	2.022 × 10^−5^	0.274	−1.996 × 10^−3^	0.959
4.	*SNORD94*	small nucleolar RNA, C/D box 94	3.480 × 10^−5^	0.441	−1.229 × 10^−3^	0.939
5.	*MALT1*	MALT1 paracaspase	1.177 × 10^−4^	0.762	−4.413 × 10^−4^	1.000
6.	*SNORD127*	small nucleolar RNA, C/D box 127	1.519 × 10^−4^	0.550	−9.698 × 10^−4^	0.959
7.	*SNORA14B*	small nucleolar RNA, H/ACA box 14B	4.364 × 10^−4^	0.672	−6.430 × 10^−4^	0.959
8.	*STMN3*	stathmin 3	4.598 × 10^−4^	0.603	−8.031 × 10^−4^	0.939
9.	*TCP11L2*	t-complex 11 like 2	7.061 × 10^−4^	0.689	−6.071 × 10^−4^	1.000
10.	*SNORA60*	small nucleolar RNA, H/ACA box 60	9.366 × 10^−4^	0.641	−7.161 × 10^−4^	0.959

AAA—abdominal aortic aneurysm, CVD—chronic venous disease, LEAD—lower extremities arterial disease, ROC—receiver operating characteristics, UVE-PLS—Uninformative Variable Elimination by Partial Least Squares.

**Table 3:** Correlation analysis between characteristics of studied groups (age, BMI, creatinine level) and expression of 21, 58 and 10 genes identified as unique for LEAD vs. AAA, LEAD vs. CVD and AAA vs. CVD comparisons, respectively. The table presents genes correlated with statistical significance (Benjamini–Hochberg FDR adjusted *p* < 0.05) and with the absolute value of Spearman correlation coefficient R ≥ 0.6 (the entire correlation results are provided in Supplementary File 2.

Comparison	Age			BMI			Creatinine		
	Gene Symbol	R	*p*	Gene Symbol	R	*p*	Gene Symbol	R	*p*
**LEAD vs. AAA**	none			none			*POLR2A*	−0.65	2.91 × 10^−3^
							*ZNF592*	−0.62	5.25 × 10^−3^
							*TRAPPC12*	−0.60	7.01 × 10^−3^
**LEAD vs. CVD**	*PSME2P2*	0.69	1.27 × 10^−3^	*TECPR1*	−0.76	2.25 × 10^−4^	*RP11−262D11.2*	0.72	6.68 × 10^−4^
	*FCGR3B*	0.69	1.35 × 10^−3^	*PIDD*	−0.75	2.97 × 10^−4^	*SRRM1P3*	0.70	9.06 × 10^−4^
	*API5P1*	0.67	1.83 × 10^−3^	*PSME1*	0.67	2.01 × 10^−3^	*SDCBPP2*	0.70	9.66 × 10^−4^
	*ACTR3P2*	0.65	2.80 × 10^−3^	*D2HGDH*	−0.66	2.42 × 10^−3^	*ARL6IP1*	0.67	1.83 × 10^−3^
	*CDC42P6*	0.64	3.80 × 10^−3^	*HSP90B3P*	0.65	3.04 × 10^−3^	*HNRNPA1P7*	0.67	1.83 × 10^−3^
	*HSP90B2P*	0.63	4.24 × 10^−3^	*PPP6R2*	−0.64	3.39 × 10^−3^	*API5P1*	0.67	1.94 × 10^−3^
	*PIDD1*	−0.62	4.70 × 10^−3^	*EIF3C*	0.62	3.41 × 10^−3^	*AC104651.2*	0.66	2.39 × 10^−3^
	*SGSM3*	−0.62	4.79 × 10^−3^	*HSPA9P1*	0.60	6.72 × 10^−3^	*EIF3FP3*	0.65	2.85 × 10^−3^
	*CAP1P2*	0.62	4.89 × 10^−3^				*RP11-286H14.4*	0.65	2.88 × 10^−3^
	*RP11-6B6.3*	0.62	5.08 × 10^−3^				*CTNNA1P1*	0.64	3.25 × 10^−3^
	*RP11-490H24.5*	0.62	5.14 × 10^−3^				*DYNC1I2P1*	0.64	3.54 × 10^−3^
	*RP13-104F24.3*	0.61	5.55 × 10^−3^				*CTB-52I2.4*	0.62	5.49 × 10^−3^
	*CTB-52I2.4*	0.61	5.65 × 10^−3^						
	*HSP90B3P*	0.61	5.77 × 10^−3^						
	*CTNNA1P1*	0.60	6.83 × 10^−3^						
**AAA vs. CVD**	*SNORD64*	−0.68	1.40 × 10^−3^	none			none		
	*STMN3*	−0.66	2.20 × 10^−3^						
	*MIR150*	−0.65	2.99 × 10^−3^						
	*MALT1*	−0.63	4.39 × 10^−3^						

None—no genes meeting selection criteria (R ≥ 0.6 and corrected *p* < 0.05), R—Spearman correlation coefficient, BMI—body mass index, AAA—abdominal aortic aneurysm, CVD—chronic venous disease, LEAD—lower extremities arterial disease.

**Table 4:** Relationships between categorical characteristics of study subjects (smoking and hypertension status, medication with statins, acetylsalicylic acid and beta-adrenergic blockers) and expression of 89 genes selected from LEAD vs. AAA, LEAD vs. CVD and AAA vs. CVD comparisons. The table presents genes with statistically significant relationship (Benjamini–Hochberg FDR adjusted *p* < 0.05) obtained from two-sided Mann–Whitney U test (the entire results are provided in Supplementary File 2).

Comparison	Hypertension Status		Statins Medication		Acetylsalicylic Acid					
	Gene Symbol	*p*	Gene Symbol	*p*	Gene Symbol	*p*	Gene Symbol	*p*	Gene Symbol	*p*
**LEAD vs. AAA**	none		none		none					
**LEAD vs. CVD**	*GLI4*	8.09 × 10^−3^	*FAM167A*	2.05 × 10^−2^	*FCGR3B*	2.09 × 10^−3^	*SGSM3*	5.22 × 10^−3^	*D2HGDH*	1.09 × 10^−2^
	*MNDA*	3.52 × 10^−2^	*C1orf216*	4.76 × 10^−2^	*GLI4*	2.09 × 10^−3^	*CDC42P6*	6.02 × 10^−3^	*DYNC1I2P1*	1.09 × 10^−2^
					*HSP90B2P*	2.09 × 10^−3^	*EIF3C*	6.02 × 10^−3^	*EIF3FP3*	1.09 × 10^−2^
					*PSME2P2*	2.09 × 10^−3^	*EIF4A1P10*	6.02 × 10^−3^	*PGDP1*	1.09 × 10^−2^
					*TECPR1*	2.09 × 10^−3^	*PSME1*	6.02 × 10^−3^	*RASGRP2*	1.09 × 10^−2^
					*HSP90B3P*	2.32 × 10^−3^	*RP11-6B6.3*	6.02 × 10^−3^	*SDCBPP2*	1.09 × 10^−2^
					*PIDD1*	2.32 × 10^−3^	*ANXA2P2*	7.34 × 10^−3^	*CALM2P4*	1.38 × 10^−2^
					*RP11-490H24.5*	2.32 × 10^−3^	*CAP1P2*	7.34 × 10^−3^	*FAM167A*	1.38 × 10^−2^
					*RP13-104F24.3*	2.32 × 10^−3^	*HIP1R*	7.34 × 10^−3^	*RP11-262D11.2*	1.38 × 10^−2^
					*HSPA9P1*	3.04 × 10^−3^	*MSNP1*	7.34 × 10^−3^	*TBC1D27P*	1.38 × 10^−2^
					*PDIA3P1*	3.04 × 10^−3^	*POLRMT*	7.34 × 10^−3^	*C1orf216*	1.82 × 10^−2^
					*SETP14*	3.04 × 10^−3^	*RP11-1033A18.1*	7.34 × 10^−3^	*TSC2*	1.82 × 10^−2^
					*ACTR3P2*	3.91 × 10^−3^	*CALM2P2*	9.06 × 10^−3^	*SRRM1P3*	3.11 × 10^−2^
					*API5P1*	3.91 × 10^−3^	*CTB-52I2.4*	9.06 × 10^−3^	*AC104651.2*	3.90 × 10^−2^
					*C1QB*	3.91 × 10^−3^	*CTNNA1P1*	9.06 × 10^−3^	*CFL1P4*	3.90 × 10^−2^
					*HNRNPA1P7*	3.91 × 10^−3^	*GLUD2*	9.06 × 10^−3^	*HECTD4*	4.76 × 10^−2^
					*RP11-286H14.4*	5.22 × 10^−3^	*MNDA*	9.06 × 10^−3^	*PAM16*	4.76 × 10^−2^
					*RP11-334L9.1*	5.22 × 10^−3^	*AC078899.1*	1.09 × 10**^−2^**	*S100A10*	4.76 × 10^−2^
**AAA vs. CVD**	none		none		*TCP11L2*	6.02 × 10^−3^	*STMN3*	1.38 × 10^−2^	*MIR150*	3.11 × 10^−2^
					*MALT1*	6.02 × 10^−3^				

None—no genes meeting selection criteria (corrected *p* < 0.05), AAA—abdominal aortic aneurysm, CVD—chronic venous disease, LEAD—lower extremities arterial disease.

**Table 5:** Gene types of 21, 58 and 10 genes identified as specific for LEAD vs. AAA, LEAD vs. CVD and AAA vs. CVD comparisons, respectively. Gene types were harvested from Ensembl genome browser release 102 (http://www.ensembl.org/index.html, accessed on 20 January 2021).

Direction of Regulation	Gene Type	Gene Symbols	Number in up-/Downregulated Group of Genes	%
**LEAD vs. AAA**				
up	snoRNA	*SNORA26, SNORA72, SNORD101*, *SNORD111B, SNORD20*, *SNORD82*	6/9	66.7
	protein coding	*UFM1*	1/9	11.1
	lncRNA	*SNHG5*	1/9	11.1
	sense intronic	*SNORD91B*	1/9	11.1
down	protein coding	*POLR2A*, *EHMT1*, *TRAPPC12*, *ZNF592*, *YBX1*, *MAU2*, *GIT2*	7/12	58.3
	misc RNA	*RN7SKP208, RN7SKP286*, *RN7SKP45*, *RN7SKP7*	4/12	33.3
	lncRNA	*AC092620.2*	1/12	8.3
**LEAD vs. CVD**				
up	pseudogene	*AC078899.1*, *AC104651.2*, *ACTR3P2*, *ANXA2P2*, *API5P1*, *CALM2P2*, *CALM2P4*, *CAP1P2*, *CDC42P6*, *CFL1P4*, *CTB-52I2.4*, *CTNNA1P1*, *DYNC1I2P1*, *EIF3FP3*, *EIF4A1P10*, *HNRNPA1P7*, *HSP90B2P*, *HSP90B3P*, *HSPA9P1*, *MSNP1*, *PDIA3P1*, *PGDP1*, *PSME2P2*, *RP11-1033A18.1*, *RP11-262D11.2*, *RP11-286H14.4*, *RP11-334L9.1*, *RP11-490H24.5*, *RP11-6B6.3*, *RP13-104F24.3*, *SDCBPP2*, *SETP14*, *SRRM1P3*	33/43	76.7
	protein coding	*ARL6IP1*, *C1orf216*, *C1QB*, *EIF3C*, *FCGR3B*, *GLUD2*, *MNDA*, *PSME1*, *S100A10*, *S100A12*	10/43	23.3
down	protein coding	*D2HGDH*, *DNAH1*, *FAM167A*, *GLI4*, *HECTD4*, *HIP1R*, *PAM16*, *PIDD1*, *POLRMT*, *PPP6R2*, *RASGRP2*, *SGSM3*, *TECPR1*, *TSC2*	14/15	93.3
	pseudogene	*TBC1D27P*	1/15	6.7
**AAA vs. CVD**				
down	snoRNA	*SNORA11*, *SNORA14B*, *SNORA60*, *SNORD127*, *SNORD64*, *SNORD94*	6/10	60
	protein coding	*MALT1*, *STMN3*, *TCP11L2*	3/10	30
	miRNA	*MIR150*	1/10	10

AAA—abdominal aortic aneurysm, CVD—chronic venous disease, LEAD—lower extremities arterial disease, lncRNA—long non-coding RNA, miRNA—microRNA, misc RNA—miscellaneous RNA, snoRNA—small nucleolar RNA.

## Data Availability

The data generated for this study are openly available in FigShare repository at https://doi.org/10.6084/m9.figshare.14252897.v1.

## Supplementary Materials

Supplementary materials can be found at https://www.mdpi.com/1422-0067/22/6/3200/s1, Figure S1: Quality control of results obtained from differential gene expression analysis performed by DESeq2 package between group of 8 LEAD subjects and a group of 7 CVD subjects, Figure S2: Quality control of results obtained from differential gene expression analysis performed by DESeq2 package between group of 8 LEAD subjects and a group of 7 AAA subjects, Figure S3: Quality control of results obtained from differential gene expression analysis performed by DESeq2 package between group of 7 AAA subjects and a group of 7 CVD subjects, Figure S4: Boxplot presenting Cook’s distances of genes across samples, Figure S5: Proportions of white blood cells subpopulations in the study subjects, resulted from blood morphology analysis, Figure S6: Plots presenting the arrangement of prediction error and Partial Least Squares (PLS) components generated in UVE-PLS differential expression analysis of gene expression data, Table S1: Results of ROC analysis for 21 genes unique for the comparison of LEAD vs. AAA, 58 genes unique for the comparison of LEAD vs. CVD and 10 genes unique for the comparison of AAA vs. CVD, Table S2: Assessment of transcriptome libraries and results of primary analysis of transcriptome sequencing data carried out with Ion Torrent RNASeqAnalysis plugin v.5.0.3.0.

## Abbreviations

AAA	Abdominal Aortic Aneurysm
AMPK	AMP-activated protein kinase
BMI	Body Mass Index
CVD	Chronic Venous Disease
DAVID	Database for Annotation, Visualization and Integrated Discovery
GOBP	Gene Ontology Biological Processing
GOCC	Gene Ontology Cellular Compartment
GOMF	Gene Ontology Molecular Function
KEGG	Kyoto Encyclopedia of Genes and Genomes
LEAD	Lower Extremities Arterial Disease
miRNA	microRNA
PAD	Peripheral Arterial Disease
PBMCs	Peripheral Blood Mononuclear Cells
PCA	Principal Component Analysis
PLS	Partial Least Squares
ROC	Receiver Operating Characteristics
ROC-AUC	Area under ROC curve
snoRNA	Small nucleolar RNA
UVE-PLS	Uninformative Variable Elimination by Partial Least Squares

## Appendix A

**Table A1:** Detailed clinical characteristics of the study subjects specific for LEAD, AAA and CVD groups.

Group	Parameter	
LEAD (*n* = 8)	**Indication for treatment:**	
	Rutherford category 2	6 (75%)
	Rutherford category 3	2 (25%)
	Initial claudication distance (m)	143.75 ± 26.69 (100–180) ^1^
	Ankle-brachial index	0.658 ± 0.045 (0.59–0.72) ^1^
	Length of occlusion (cm)	13.63 ± 5.15 (7–22) ^1^
	Plaque localization:	
	Iliac artery	1 (12.5%)
	Femoral artery	6 (75%)
	Iliac and femoral artery	1 (12.5%)
AAA (*n* = 7)	**Abdominal aneurysm measurements:**	
	Maximum aneurysm diameter (cm)	6.371 ± 0.419 (5.8–7.0) ^1^
	Thrombus volume (cm^3^)	10.821 ± 2.605 (6.3–14.7) ^1^
	Aneurysm neck length (cm)	0.971 ± 0.198 (0.7–1.2) ^1^
CVD (*n* = 7)	**Signs and symptoms:**	
	Pain	2 (28.6%)
	Ankle-brachial index	0.974 ± 0.016 (0.95–0.99) ^1^
	Extended anatomical classification:	
	Great saphenous vein (above knee)	3 (42.8%)
	Great saphenous vein (below knee)	2 (28.6%)
	Small saphenous vein	2 (28.6%)
	Medication:	
	Micronized diosmin	3 (42.98)
	Preparation with vitamin C, hesperidin and *Ruscus aculeatus* extract	2 (28.6%)
	Both medications	2 (28.6%)

^1^ mean ± SD (range). LEAD—lower extremities arterial disease, AAA—abdominal aortic aneurysm, CVD—chronic venous disease.

## Appendix B

**Table A2:** General results of differential gene expression analysis performed using DESeq2 and UVE-PLS methods obtained for comparisons: LEAD vs. AAA, LEAD vs. CVD and AAA vs. CVD.

Comparison	DESeq2			UVE-PLS		Number of Genes Common for Sets of Genes Selected from DESeq2 (*p* < 0.001) and from UVE-PLS (Reliability Score ≥8)
	Number of All Differentially Expressed Genes	Number of Differentially Expressed Genes with *p* < 0.05	NUMBER of Differentially Expressed Genes with *p* < 0.001	Number of PLS Components/Iterations	Number of Informative Genes with Reliability Score ≥8	
LEAD vs. AAA	21,460	544	31	3/1000	89	21
LEAD vs. CVD	21,460	1603	87	3/1000	174	58
AAA vs. CVD	20,550	685	56	2/1000	34	10

*p*—statistical significance after correction by Benjamini–Hochberg false discovery rate, AAA—abdominal aortic aneurysm, CVD—chronic venous disease, LEAD—lower extremities arterial disease, UVE-PLS—Uninformative Variable Elimination by Partial Least Squares.
