# Supplementary material for: Identification of Transcriptomic Differences between Lower Extremities Arterial Disease, Abdominal Aortic Aneurysm and Chronic Venous Disease in Peripheral Blood Mononuclear Cells Specimens
Source: Int J Mol Sci. 2021 Mar 21;22(6):3200. doi: 10.3390/ijms22063200 (PMC8004090; doi:10.3390/ijms22063200)
Supplement: Supplementary file 1 [file ijms-22-03200-s001.zip › ijms-1113252-supplementary/SupplementaryFile1_submission.docx]

**SUPPLEMENTARY MATERIAL**

**Supplementary Figures**


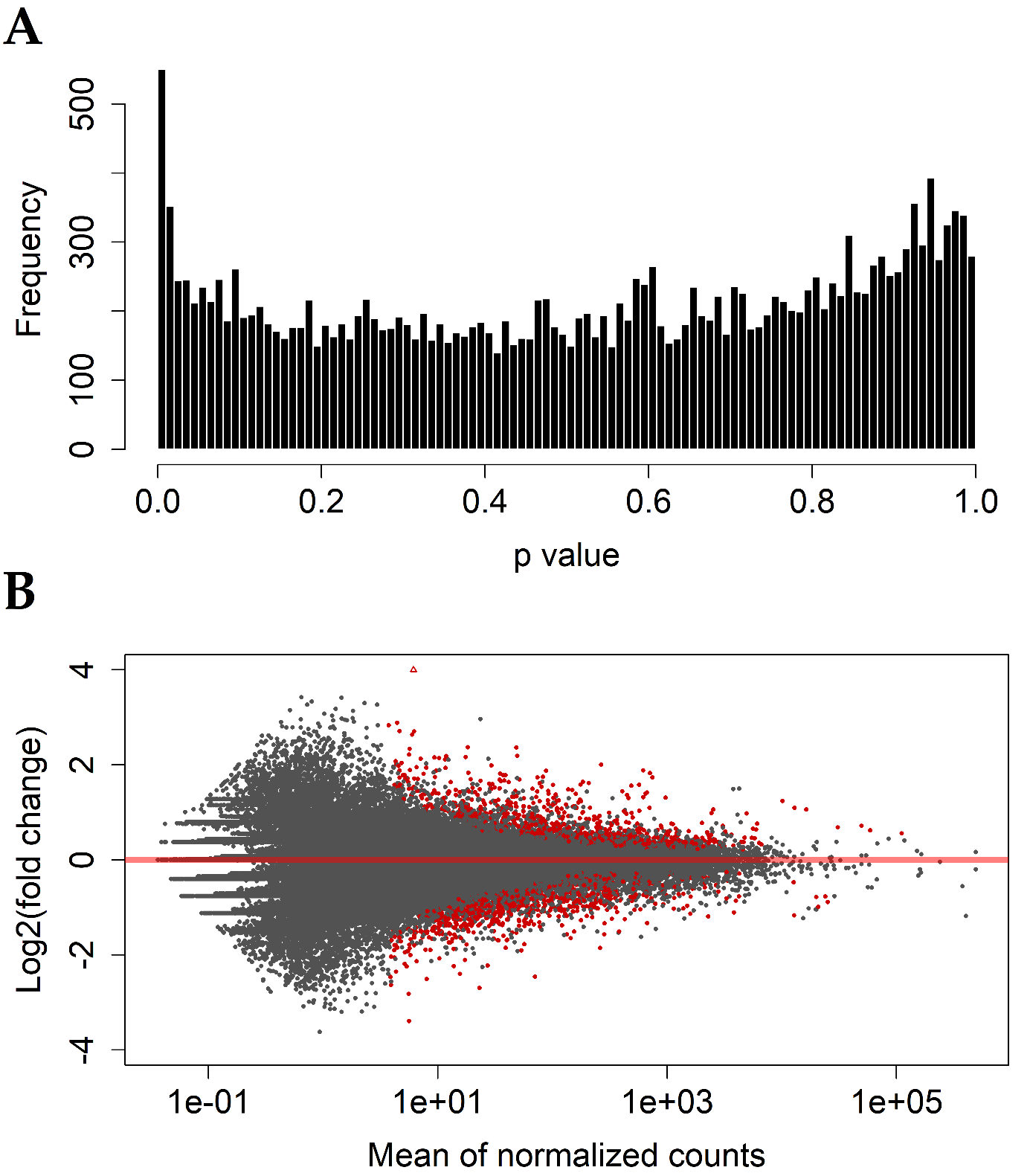


**Figure S1.** Quality control of results obtained from differential gene expression analysis performed by DESeq2 package between group of 8 LEAD subjects and a group of 7 CVD subjects. (**A**) Histogram presenting distribution of *p* values. (**B**) MA plot showing relation between log2 of fold changes of differentially expressed genes and averages of normalized counts. Genes with *p* value < 0.05 were marked as red points.


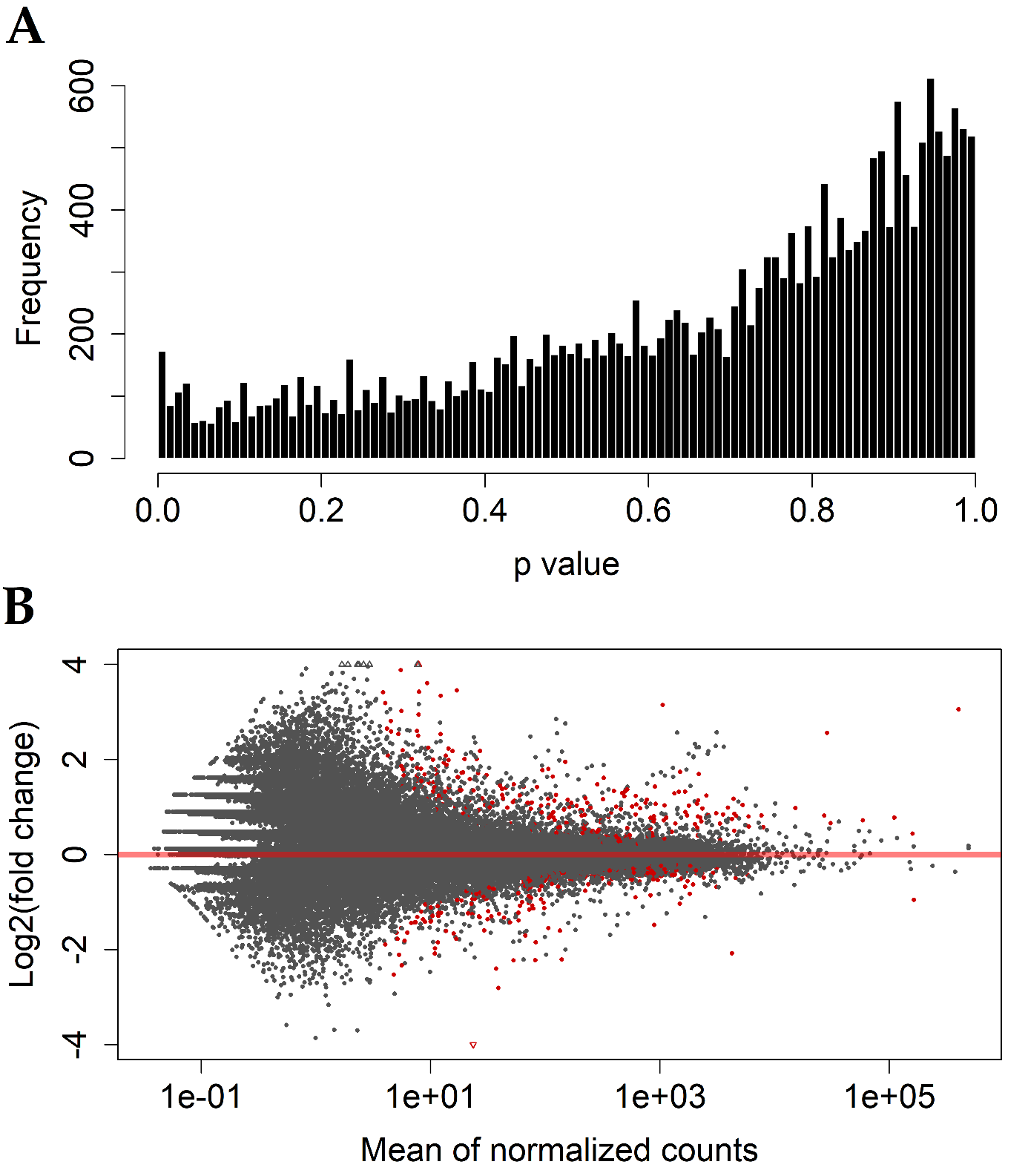


**Figure S2.** Quality control of results obtained from differential gene expression analysis performed by DESeq2 package between group of 8 LEAD subjects and a group of 7 AAA subjects. (**A**) Histogram presenting distribution of *p* values. (**B**) MA plot showing relation between log2 of fold changes of differentially expressed genes and averages of normalized counts. Genes with *p* value < 0.05 were marked as red points.


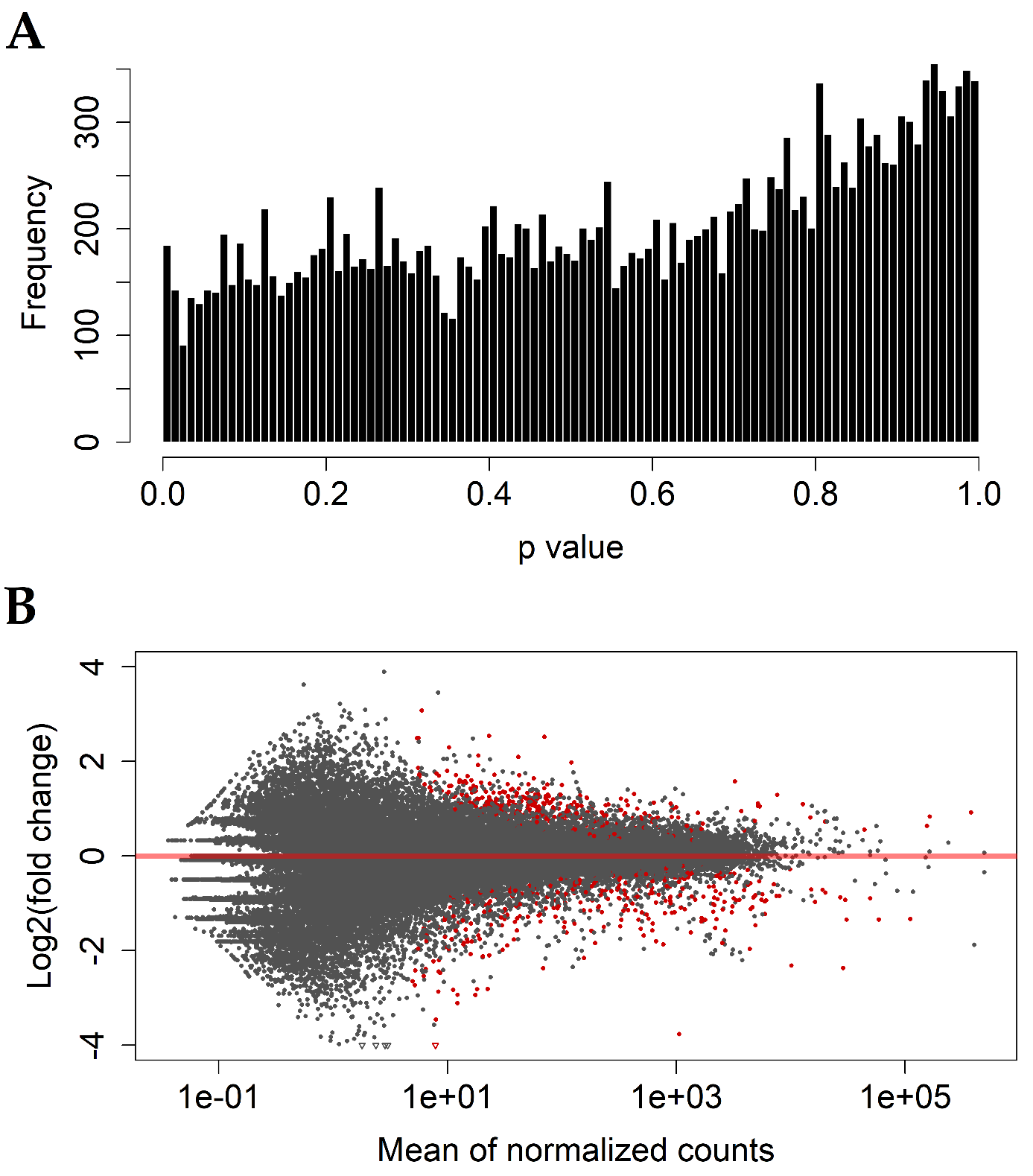


**Figure S3.** Quality control of results obtained from differential gene expression analysis performed by DESeq2 package between group of 7 AAA subjects and a group of 7 CVD subjects. (**A**) Histogram presenting distribution of *p* values. (**B**) MA plot showing relation between log2 of fold changes of differentially expressed genes and averages of normalized counts. Genes with *p* value < 0.05 were marked as red points.


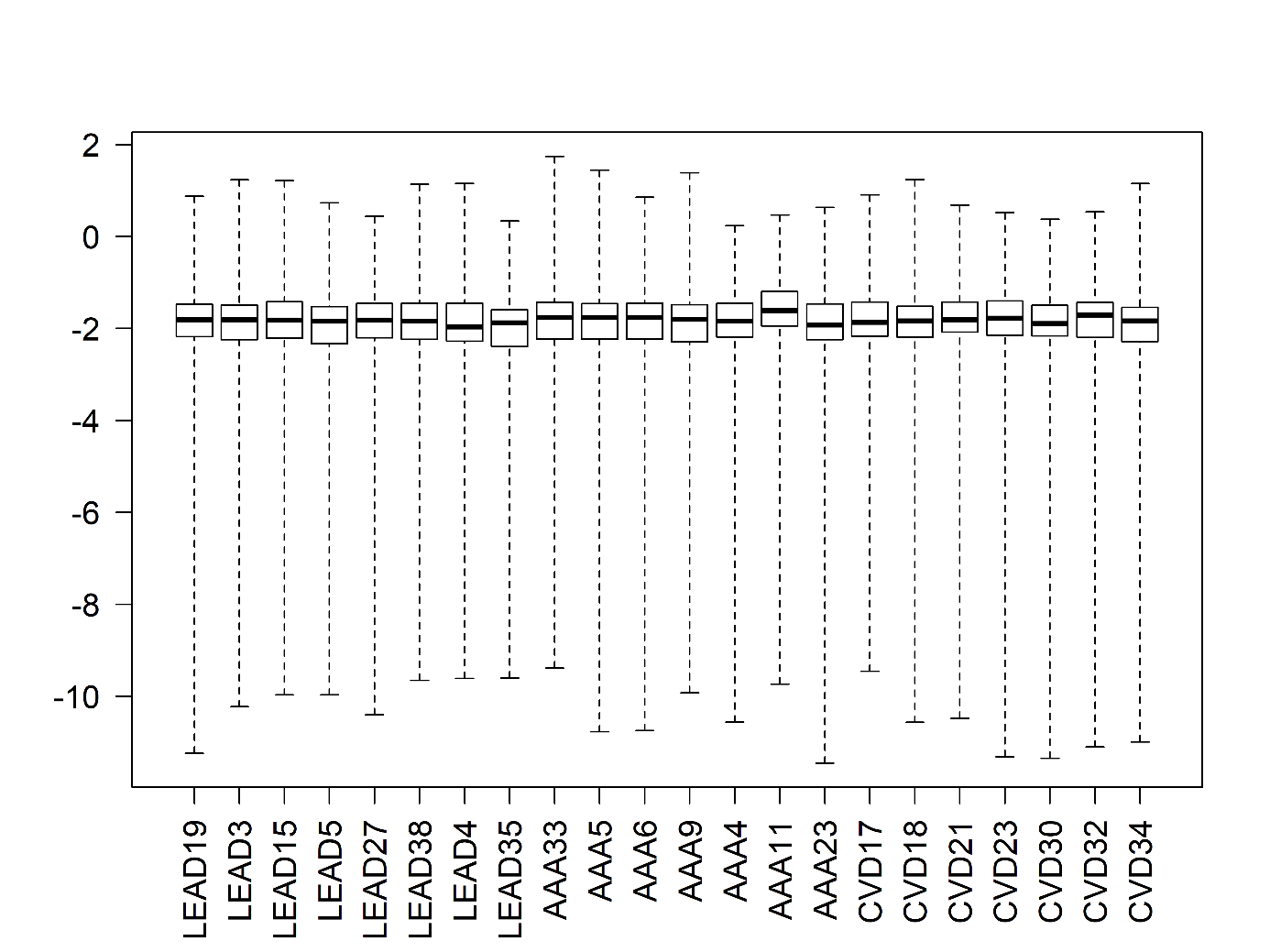


**Figure S4.** Boxplot presenting Cook’s distances of genes across samples. Whiskers define range between minimum and maximum value of Cook’s distance, boxes range between 25% and 75% quartile, horizontal lines inside boxes mark median value. LEAD - lower extremities arterial disease, AAA - abdominal aortic aneurysm, CVD - chronic venous disease.


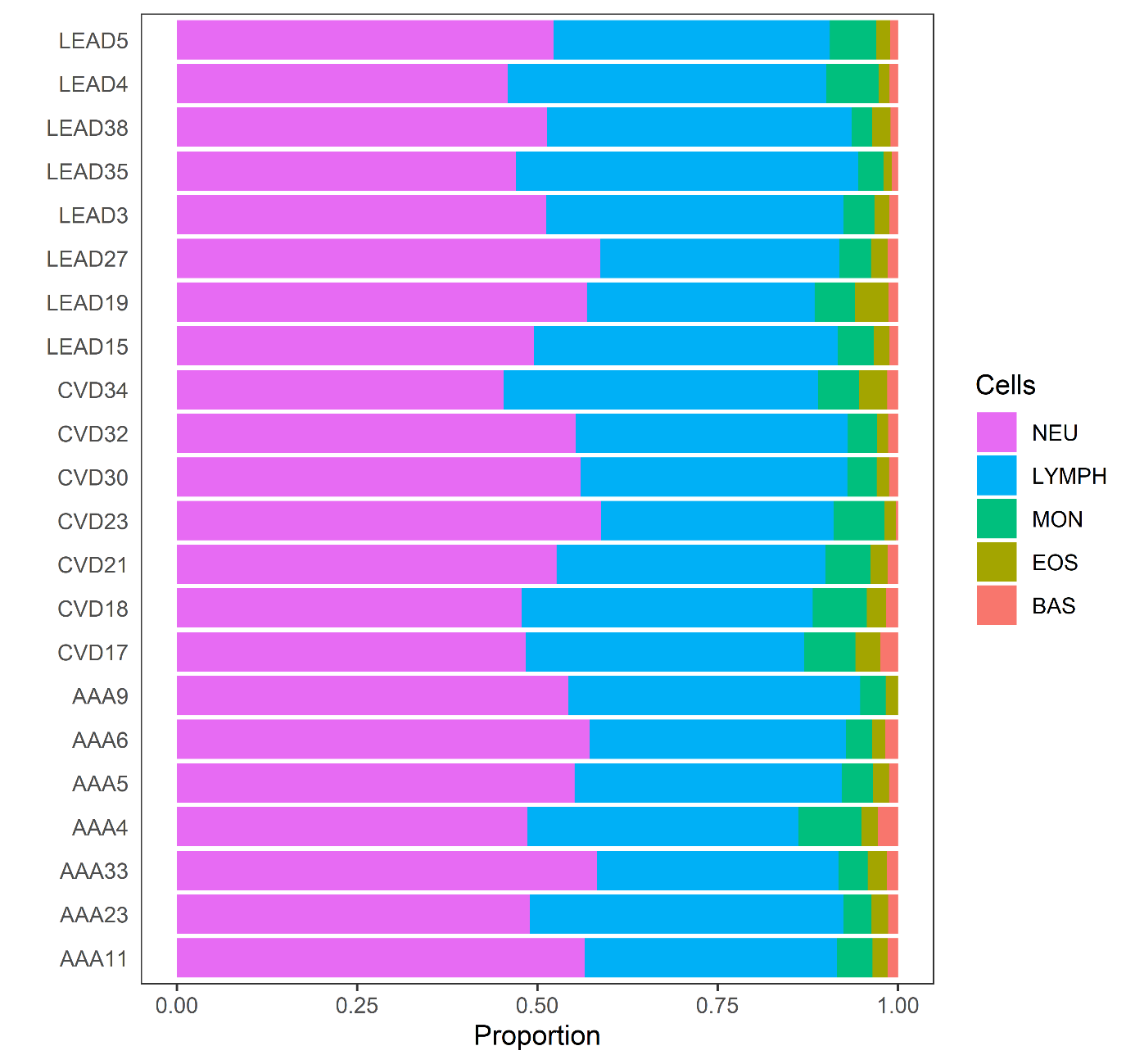


**Figure S5.** Proportions of white blood cells subpopulations in the study subjects, resulted from blood morphology analysis. NEU – neutrophils, LYMPH – lymphocytes, MON – monocytes, EOS – eosinophils, BAS – basophils, LEAD - lower extremities arterial disease, AAA - abdominal aortic aneurysm, CVD - chronic venous disease.


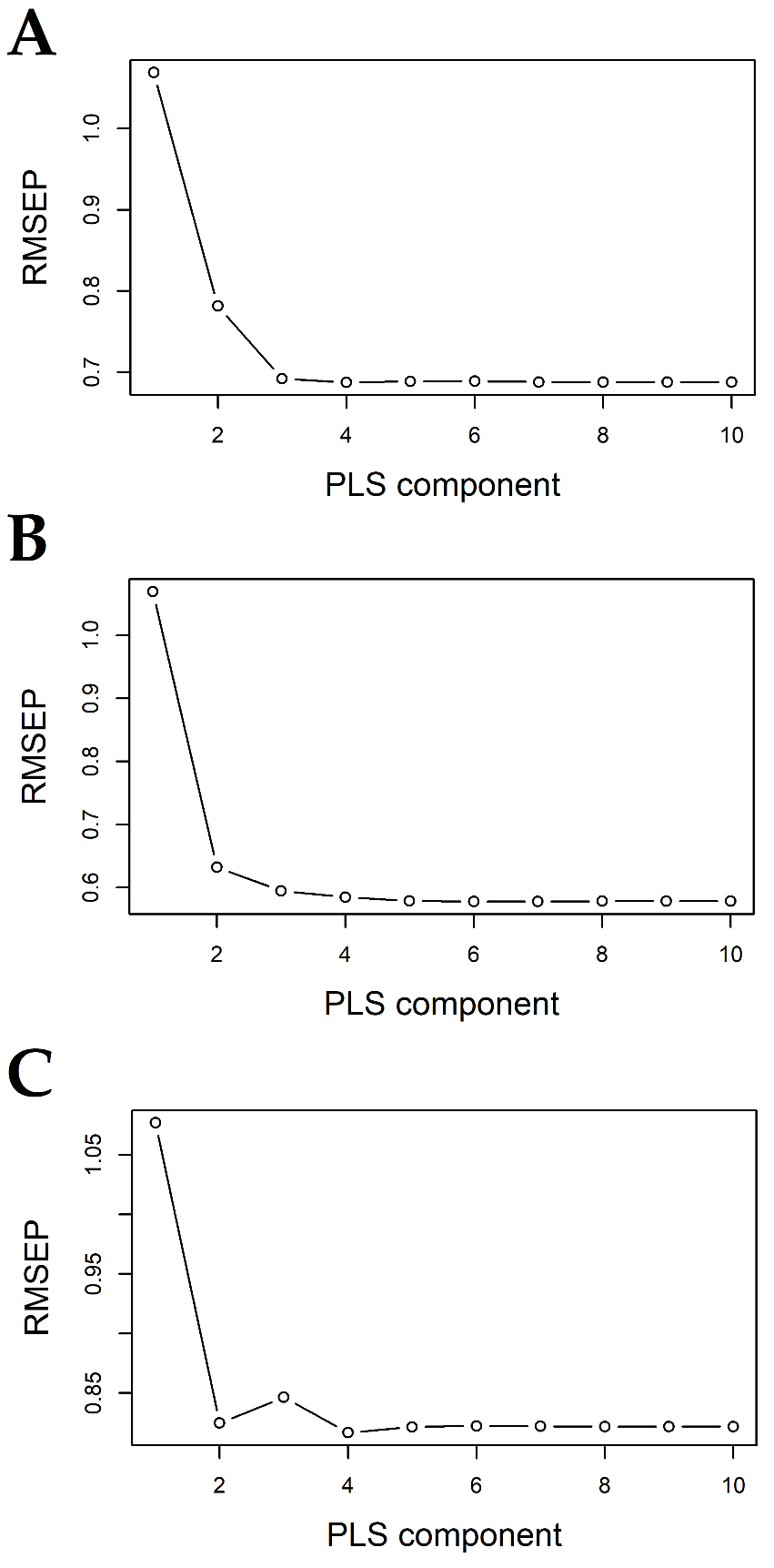


**Figure S6.** Plots presenting the arrangement of prediction error and Partial Least Squares (PLS) components generated in UVE-PLS differential expression analysis of gene expression data for following comparisons: (**A**) 8 LEAD subjects versus 7 AAA subjects, (**B**) 8 LEAD subjects versus 7 CVD subjects and (**C**) 7 AAA subjects versus 7 CVD subjects. RMSEP - Root Mean Squared Error of Prediction

**Supplementary Tables**

**Table S1.** Results of ROC analysis for 21 genes unique for the comparison of LEAD vs AAA, 58 genes unique for the comparison of LEAD vs CVD and 10 genes unique for the comparison of AAA vs CVD.

| **Gene symbol** | **ROC-AUC^1^** | **Threshold** | **Specificity** | **Sensitivity** | **Accuracy** | **Positive Predictive Value** | **Negative Predictive Value** |
| --- | --- | --- | --- | --- | --- | --- | --- |
| **LEAD vs AAA** | | | | | | | |
| *AC092620.2* | 1.000 | 4.719 | 1.000 | 1.000 | 1.000 | 1.000 | 1.000 |
| *EHMT1* | 1.000 | 10.400 | 1.000 | 1.000 | 1.000 | 1.000 | 1.000 |
| *GIT2* | 1.000 | 10.764 | 1.000 | 1.000 | 1.000 | 1.000 | 1.000 |
| *MAU2* | 0.982 | 10.448 | 0.875 | 1.000 | 0.933 | 0.875 | 1.000 |
| *POLR2A* | 1.000 | 11.563 | 1.000 | 1.000 | 1.000 | 1.000 | 1.000 |
| *RN7SKP208* | 0.982 | 3.833 | 0.875 | 1.000 | 0.933 | 0.875 | 1.000 |
| *RN7SKP286* | 0.964 | 5.281 | 1.000 | 0.857 | 0.933 | 1.000 | 0.889 |
| *RN7SKP45* | 0.982 | 5.206 | 0.875 | 1.000 | 0.933 | 0.875 | 1.000 |
| *RN7SKP7* | 1.000 | 2.498 | 1.000 | 1.000 | 1.000 | 1.000 | 1.000 |
| *SNHG5* | 1.000 | 11.747 | 1.000 | 1.000 | 1.000 | 1.000 | 1.000 |
| *SNORA26* | 1.000 | 11.083 | 1.000 | 1.000 | 1.000 | 1.000 | 1.000 |
| *SNORA72* | 0.964 | 10.729 | 1.000 | 0.857 | 0.933 | 1.000 | 0.889 |
| *SNORD101* | 0.964 | 9.168 | 0.875 | 1.000 | 0.933 | 0.875 | 1.000 |
| *SNORD111B* | 1.000 | 8.606 | 1.000 | 1.000 | 1.000 | 1.000 | 1.000 |
| *SNORD20* | 1.000 | 10.334 | 1.000 | 1.000 | 1.000 | 1.000 | 1.000 |
| *SNORD82* | 1.000 | 10.794 | 1.000 | 1.000 | 1.000 | 1.000 | 1.000 |
| *SNORD91B* | 1.000 | 10.298 | 1.000 | 1.000 | 1.000 | 1.000 | 1.000 |
| *TRAPPC12* | 0.982 | 9.566 | 0.875 | 1.000 | 0.933 | 0.875 | 1.000 |
| *UFM1* | 1.000 | 8.112 | 1.000 | 1.000 | 1.000 | 1.000 | 1.000 |
| *YBX1* | 0.982 | 10.653 | 0.875 | 1.000 | 0.933 | 0.875 | 1.000 |
| *ZNF592* | 1.000 | 10.335 | 1.000 | 1.000 | 1.000 | 1.000 | 1.000 |
| **LEAD vs CVD** | | | | | | | |
| *AC078899.1* | 0.982 | 7.163 | 0.875 | 1.000 | 0.933 | 0.875 | 1.000 |
| *AC104651.2* | 0.946 | 3.474 | 0.875 | 1.000 | 0.933 | 0.875 | 1.000 |
| *ACTR3P2* | 1.000 | 5.781 | 1.000 | 1.000 | 1.000 | 1.000 | 1.000 |
| *ANXA2P2* | 1.000 | 7.898 | 1.000 | 1.000 | 1.000 | 1.000 | 1.000 |
| *API5P1* | 1.000 | 4.842 | 1.000 | 1.000 | 1.000 | 1.000 | 1.000 |
| *ARL6IP1* | 1.000 | 7.944 | 1.000 | 1.000 | 1.000 | 1.000 | 1.000 |
| *C1QB* | 0.964 | 5.331 | 1.000 | 0.857 | 0.933 | 1.000 | 0.889 |
| *C1orf216* | 0.982 | 6.690 | 0.875 | 1.000 | 0.933 | 0.875 | 1.000 |
| *CALM2P2* | 1.000 | 5.594 | 1.000 | 1.000 | 1.000 | 1.000 | 1.000 |
| *CALM2P4* | 1.000 | 4.677 | 1.000 | 1.000 | 1.000 | 1.000 | 1.000 |
| *CAP1P2* | 1.000 | 7.058 | 1.000 | 1.000 | 1.000 | 1.000 | 1.000 |
| *CDC42P6* | 1.000 | 7.319 | 1.000 | 1.000 | 1.000 | 1.000 | 1.000 |
| *CFL1P4* | 1.000 | 4.725 | 1.000 | 1.000 | 1.000 | 1.000 | 1.000 |
| *CTB-52I2.4* | 0.982 | 5.920 | 0.875 | 1.000 | 0.933 | 0.875 | 1.000 |
| *CTNNA1P1* | 0.982 | 4.897 | 0.875 | 1.000 | 0.933 | 0.875 | 1.000 |
| *D2HGDH* | 0.964 | 8.477 | 0.875 | 1.000 | 0.933 | 0.875 | 1.000 |
| *DNAH1* | 1.000 | 11.162 | 1.000 | 1.000 | 1.000 | 1.000 | 1.000 |
| *DYNC1I2P1* | 1.000 | 5.650 | 1.000 | 1.000 | 1.000 | 1.000 | 1.000 |
| *EIF3C* | 1.000 | 8.139 | 1.000 | 1.000 | 1.000 | 1.000 | 1.000 |
| *EIF3FP3* | 0.964 | 7.305 | 0.875 | 1.000 | 0.933 | 0.875 | 1.000 |
| *EIF4A1P10* | 1.000 | 7.640 | 1.000 | 1.000 | 1.000 | 1.000 | 1.000 |
| *FAM167A* | 0.982 | 4.170 | 0.875 | 1.000 | 0.933 | 0.875 | 1.000 |
| *FCGR3B* | 1.000 | 6.666 | 1.000 | 1.000 | 1.000 | 1.000 | 1.000 |
| *GLI4* | 1.000 | 7.844 | 1.000 | 1.000 | 1.000 | 1.000 | 1.000 |
| *GLUD2* | 0.982 | 6.653 | 0.875 | 1.000 | 0.933 | 0.875 | 1.000 |
| *HECTD4* | 0.982 | 11.135 | 0.875 | 1.000 | 0.933 | 0.875 | 1.000 |
| *HIP1R* | 1.000 | 9.018 | 1.000 | 1.000 | 1.000 | 1.000 | 1.000 |
| *HNRNPA1P7* | 1.000 | 7.744 | 1.000 | 1.000 | 1.000 | 1.000 | 1.000 |
| *HSP90B2P* | 1.000 | 6.454 | 1.000 | 1.000 | 1.000 | 1.000 | 1.000 |
| *HSP90B3P* | 1.000 | 5.543 | 1.000 | 1.000 | 1.000 | 1.000 | 1.000 |
| *HSPA9P1* | 1.000 | 5.831 | 1.000 | 1.000 | 1.000 | 1.000 | 1.000 |
| *MNDA* | 1.000 | 10.420 | 1.000 | 1.000 | 1.000 | 1.000 | 1.000 |
| *MSNP1* | 1.000 | 8.891 | 1.000 | 1.000 | 1.000 | 1.000 | 1.000 |
| *PAM16* | 0.982 | 6.055 | 0.875 | 1.000 | 0.933 | 0.875 | 1.000 |
| *PDIA3P1* | 1.000 | 7.307 | 1.000 | 1.000 | 1.000 | 1.000 | 1.000 |
| *PGDP1* | 0.982 | 6.038 | 0.875 | 1.000 | 0.933 | 0.875 | 1.000 |
| *PIDD1* | 1.000 | 7.673 | 1.000 | 1.000 | 1.000 | 1.000 | 1.000 |
| *POLRMT* | 1.000 | 8.893 | 1.000 | 1.000 | 1.000 | 1.000 | 1.000 |
| *PPP6R2* | 1.000 | 10.077 | 1.000 | 1.000 | 1.000 | 1.000 | 1.000 |
| *PSME1* | 1.000 | 9.921 | 1.000 | 1.000 | 1.000 | 1.000 | 1.000 |
| *PSME2P2* | 1.000 | 5.480 | 1.000 | 1.000 | 1.000 | 1.000 | 1.000 |
| *RASGRP2* | 0.964 | 11.716 | 1.000 | 0.857 | 0.933 | 1.000 | 0.889 |
| *RP11-1033A18.1* | 1.000 | 7.335 | 1.000 | 1.000 | 1.000 | 1.000 | 1.000 |
| *RP11-262D11.2* | 0.946 | 7.569 | 0.875 | 1.000 | 0.933 | 0.875 | 1.000 |
| *RP11-286H14.4* | 1.000 | 5.763 | 1.000 | 1.000 | 1.000 | 1.000 | 1.000 |
| *RP11-334L9.1* | 0.982 | 4.869 | 0.875 | 1.000 | 0.933 | 0.875 | 1.000 |
| *RP11-490H24.5* | 1.000 | 4.779 | 1.000 | 1.000 | 1.000 | 1.000 | 1.000 |
| *RP11-6B6.3* | 1.000 | 5.128 | 1.000 | 1.000 | 1.000 | 1.000 | 1.000 |
| *RP13-104F24.3* | 0.982 | 4.936 | 0.875 | 1.000 | 0.933 | 0.875 | 1.000 |
| *S100A10* | 1.000 | 8.987 | 1.000 | 1.000 | 1.000 | 1.000 | 1.000 |
| *S100A12* | 0.946 | 7.929 | 0.875 | 1.000 | 0.933 | 0.875 | 1.000 |
| *SDCBPP2* | 1.000 | 4.838 | 1.000 | 1.000 | 1.000 | 1.000 | 1.000 |
| *SETP14* | 1.000 | 6.435 | 1.000 | 1.000 | 1.000 | 1.000 | 1.000 |
| *SGSM3* | 1.000 | 9.676 | 1.000 | 1.000 | 1.000 | 1.000 | 1.000 |
| *SRRM1P3* | 1.000 | 4.943 | 1.000 | 1.000 | 1.000 | 1.000 | 1.000 |
| *TBC1D27P* | 1.000 | 5.357 | 1.000 | 1.000 | 1.000 | 1.000 | 1.000 |
| *TECPR1* | 1.000 | 10.259 | 1.000 | 1.000 | 1.000 | 1.000 | 1.000 |
| *TSC2* | 1.000 | 10.149 | 1.000 | 1.000 | 1.000 | 1.000 | 1.000 |
| **AAA vs CVD** | | | | | | | |
| *MALT1* | 1.000 | 9.338 | 1.000 | 1.000 | 1.000 | 1.000 | 1.000 |
| *MIR150* | 0.959 | 9.310 | 1.000 | 0.857 | 0.929 | 1.000 | 0.875 |
| *SNORA11* | 0.980 | 10.284 | 0.857 | 1.000 | 0.929 | 0.875 | 1.000 |
| *SNORA14B* | 0.959 | 12.025 | 1.000 | 0.857 | 0.929 | 1.000 | 0.875 |
| *SNORA60* | 0.959 | 9.575 | 1.000 | 0.857 | 0.929 | 1.000 | 0.875 |
| *SNORD127* | 0.959 | 9.724 | 1.000 | 0.857 | 0.929 | 1.000 | 0.875 |
| *SNORD64* | 0.959 | 7.597 | 1.000 | 0.857 | 0.929 | 1.000 | 0.875 |
| *SNORD94* | 0.939 | 10.872 | 1.000 | 0.857 | 0.929 | 1.000 | 0.875 |
| *STMN3* | 0.939 | 8.429 | 1.000 | 0.857 | 0.929 | 1.000 | 0.875 |
| *TCP11L2* | 1.000 | 9.228 | 1.000 | 1.000 | 1.000 | 1.000 | 1.000 |
| ^1^Area under ROC curve, AAA abdominal aortic aneurysm, CVD - chronic venous disease, LEAD - lower extremities arterial disease. | | | | | | | |

**Table S2.** Assessment of transcriptome libraries and results of primary analysis of transcriptome sequencing data carried out with Ion Torrent RNASeqAnalysis plugin v.5.0.3.0. The table presents mean values ± SD (standard deviation) of parameters describing results obtained in lower extremities arterial disease (LEAD) group, abdominal aortic aneurysm (AAA) group and chronic venous disease (CVD) group.

| **Parameter** | **LEAD** | | **AAA** | | **CVD** | |
| --- | --- | --- | --- | --- | --- | --- |
|  | **Mean** | **SD** | **Mean** | **SD** | **Mean** | **SD** |
| Molar concentration of libraries (pM) | 95,212.500 | 17,470.582 | 76,306.857 | 28,426.815 | 67,485.714 | 11,269.195 |
| Percentage of 50-160 bp^1^ fragments in libraries | 11.625 | 4.406 | 9.143 | 3.132 | 18.000 | 6.377 |
| Ion Sphere Particles enrichment quality control | 25.875 | 4.155 | 23.429 | 5.623 | 20.857 | 2.673 |
| Total reads | 36,090,931.125 | 3,615,078.436 | 42,124,779.000 | 4,716,653.228 | 36,387,995.000 | 8,751,010.705 |
| Aligned reads | 34,699,765.000 | 3,698,337.919 | 41,040,209.714 | 4,641,732.662 | 35,062,884.286 | 8,106,494.697 |
| Percent aligned reads | 96.10% | 1.75% | 97.41% | 0.56% | 96.55% | 1.70% |
| Mean read length | 109.588 | 11.397 | 118.471 | 8.171 | 99.429 | 13.155 |
| Genes Detected | 15,073.625 | 5,443.510 | 18,442.429 | 1,171.712 | 17,078.000 | 824.740 |
| Isoforms Detected | 49,239.250 | 4,844.657 | 53,670.000 | 6,427.859 | 52,125.714 | 5,289.771 |
| Reads mapped to genes | 12,325,052.500 | 2,786,343.742 | 17,400,523.714 | 4,758,415.420 | 12,116,282.143 | 3,279,708.873 |
| Genes with 1+ reads | 27,804.375 | 1,214.114 | 31,706.143 | 1,447.337 | 30,568.571 | 1,429.339 |
| Genes with 10+ reads | 15,073.625 | 5,443.510 | 18,299.571 | 1,056.050 | 17,078.000 | 824.740 |
| Genes with 100+ reads | 9,298.750 | 939.011 | 10,489.857 | 1,107.984 | 9,399.000 | 794.113 |
| Genes with 1000+ reads | 1,917.000 | 530.814 | 2,878.143 | 843.161 | 1,968.429 | 589.165 |
| Genes with 10000+ reads | 86.250 | 18.367 | 141.286 | 44.676 | 87.714 | 27.572 |
| Total base reads | 3,966,010,785 | 674,247,856 | 4,990,571,320 | 677,630,961 | 3,630,403,514 | 1,003,572,361 |
| Total aligned bases | 3,185,293,446 | 529,237,373 | 4,105,400,866 | 641,514,394 | 3,115,385,905 | 859,935,808 |
| Percent aligned bases | 80.39% | 3.45% | 82.20% | 4.71% | 85.94% | 1.90% |
| Percent coding bases | 14.80% | 3.85% | 20.05% | 5.74% | 15.63% | 5.36% |
| Percent UTR^2^ bases | 27.21% | 5.59% | 32.94% | 8.27% | 29.20% | 7.95% |
| Percent ribosomal bases | 5.93% | 1.60% | 3.19% | 0.92% | 4.48% | 1.25% |
| Percent intronic bases | 37.90% | 4.59% | 34.88% | 6.61% | 38.06% | 5.86% |
| Percent intergenic bases | 15.13% | 5.39% | 9.47% | 7.26% | 13.19% | 6.69% |
| Strand balance | 0.505 | 0.010 | 0.523 | 0.009 | 0.507 | 0.010 |
| ^1^base pair; ^2^Untranslated Region | | |  |  |  |  |
